# EZH2 reduction is an essential mechanoresponse for the maintenance of super-enhancer polarization against compressive stress in human periodontal ligament stem cells

**DOI:** 10.1038/s41419-020-02963-3

**Published:** 2020-09-15

**Authors:** Qian Li, Xiwen Sun, Yunyi Tang, Yanan Qu, Yanheng Zhou, Yu Zhang

**Affiliations:** 1 Department of Orthodontics, Peking University School and Hospital of Stomatology, National Engineering Laboratory for Digital and Material Technology of Stomatology, Beijing Key Laboratory of Digital Stomatology, Beijing, China; 2 Department of Biochemistry and Molecular Biology, School of Basic Medical Sciences, Peking University Health Science Center, Beijing, China

**Keywords:** Epigenetics, Mesenchymal stem cells

## Abstract

Despite the ubiquitous mechanical cues at both spatial and temporal dimensions, cell identities and functions are largely immune to the everchanging mechanical stimuli. To understand the molecular basis of this epigenetic stability, we interrogated compressive force-elicited transcriptomic changes in mesenchymal stem cells purified from human periodontal ligament (PDLSCs), and identified H3K27me3 and E2F signatures populated within upregulated and weakly downregulated genes, respectively. Consistently, expressions of several E2F family transcription factors and EZH2, as core methyltransferase for H3K27me3, decreased in response to mechanical stress, which were attributed to force-induced redistribution of RB from nucleoplasm to lamina. Importantly, although epigenomic analysis on H3K27me3 landscape only demonstrated correlating changes at one group of mechanoresponsive genes, we observed a genome-wide destabilization of super-enhancers along with aberrant EZH2 retention. These super-enhancers were tightly bounded by H3K27me3 domain on one side and exhibited attenuating H3K27ac deposition and flattening H3K27ac peaks along with compensated EZH2 expression after force exposure, analogous to increased H3K27ac entropy or decreased H3K27ac polarization. Interference of force-induced EZH2 reduction could drive actin filaments dependent spatial overlap between EZH2 and super-enhancers and functionally compromise the multipotency of PDLSC following mechanical stress. These findings together unveil a specific contribution of EZH2 reduction for the maintenance of super-enhancer stability and cell identity in mechanoresponse.

## Introduction

Cell mechanics plays important roles in tissue morphogenesis and regeneration through shaping the geometric organization of tissues and driving the movement and interaction of organelles and cells^1–3^, and it is more evident that the understanding of molecular basis for mechanoresponse is one of the central topics in mechanobiology and tissue engineering^3^. Interestingly, although the origination of mechanical stress is diverse and complex under specific microenvironment, a conserved set of protein machineries exist to perform force transmission in cells^1^. Among them, intercellular adhesions and adhesion complexes connecting extracellular matrix and plasma membrane are at the front-line for sensing mechanical strains^1,2,4^. These signals are then transmitted to cytoskeleton, and mechanical forces rely on molecular motors, such as myosin, to alter the contractility and organization of actin-myosin networks and to induce their translational or rotational movements^1,2,4^. Such physical responses are important for the target cells to make changes in size, shape, orientation, position or other geometric properties upon receiving mechanical stimuli^2^, and hence are critically implicated in the self-organization and morphogenesis of tissues across multiple scales^2,3^.

In addition to the recognition of cytoskeleton as central mechanical transducers within cytoplasm, nucleus is emerging as mechanosensor in the tensegrity model to explain the quick propagation of mechanical signals from cell membrane to distant sites^5,6^. Lamin filaments at the nuclear envelope provide major support for the structural and mechanical properties of nucleus^5,7^. The LINC (Linkers of the nucleoskeleton to the cytoskeleton) complex, consisting of Nesprin family of KASH domain proteins at the outer nuclear membrane, and SUN domain proteins SUN1 and SUN2 at the inner nuclear membrane (INM), establishes a direct physical bridge between lamina and cytoskeleton through SUN-KASH domain association^7^, therefore functionally mediating the mechanotransduction initiated at ECM or adhesion complex to the nucleus. Increasing evidence further suggests that nuclear actin polymerization and reorganization involving filamentous actin (F-actin), actin-related protein-2/3 (ARP2/3) complex, and Myosin family of motor proteins, could act downstream of LINC complex^8^ and participate in relocalization of heterochromatin break^9^, long-range chromosome motion^10^, high order chromatin structure formation and gene transcription regulation^10^.

Permanent alternations of mechanically stressed cells may also be manifested in their changes in proliferation, growth or even fate determination^2,6^. In particular, cell fate specification and lineage commitment regulated by mechanical force at longer timescale require the cells to “memorize” their identities. Epigenetic machineries capable of storing such information in the form of histone and DNA modifications, nucleosome positioning, and chromatin structure, could fulfill this task^11,12^. Polycomb repressive complex 2 (PRC2), composed by EZH2 (Enhancer of zeste homolog 2), SUZ12, and EED, is an evolutionally conserved cellular memory module for epigenetic inheritance across mitosis and meiosis^13^. This remarkable biological function has a biochemistry root, with EZH2 as the core methyltransferase component catalyzing trimethylation of lysine 27 on histone H3 (H3K27me3)^14^. Interestingly, in contrast to the sequence-specific transcription factor, PRC2 and its H3K27me3 product are not required for the specification of a fixed cell fate but for the maintenance of contemporary identity^15^. Super-enhancer (SE) is a recently identified class of regulatory element^16,17^, defined by unusually strong enrichment of P300-catalyzed H3K27ac indicative of active promoter or enhancer^18^, and dense occupancy of master transcription factors and Mediator components^16,17^. Such cluster of enhancers in close genomic proximity, showing exceptional vulnerability to enhancer perturbation and highly cooperative properties associated with epigenetic modification-dependent multivalency^19^, could functionally drive the expression of cell identity genes at high level, and thus is critically implicated in normal development and diseases^16^.

Periodontal ligament stem cells (PDLSCs) are dental mesenchymal progenitor cells extracted from periodontal ligament^20^. PDLSCs could be easily harvested, and they possess multipotency to give rise to bone, cartilage and adipose tissues in proper differentiation media^21^. These advantages make PDLSC an ideal mesenchymal stem cell (MSC) source for the treatment of bone and cartilages disorders in regenerative medicine^21^. In particular, PDLSCs have a unique potential to generate cementum/PDL-like tissue when ectopically transplanted in animals, thus holding the promise for periodontal reconstruction^20^. Mechanical cues are critically involved in the fate specification of MSC. For example, lineage commitment of bone marrow MSCs into either adipocytes or osteoblasts was regulated by mechanical stress in a dose-dependent manner^22^, and purely cyclic stretching stimulations were sufficient to induce the mesodermal cardiac differentiation of embryonic stem cells in a 3D magnetic tissue stretcher^23^.

Although it is well recognized that mechanics contributes to long timescale cellular activities such as growth and fate determination, and that epigenetics plays key roles in coordinating these programs, the functional interaction between mechanical stress and epigenetics, especially the genome-wide views of their interplay remain elusive. In this study, we used the mechanosensitive PDLSC as our model system to explore mechanical force-induced transcriptomic dynamics. This analysis combined with other functional experiments and epigenomic approaches, identified EZH2 reduction as the key epigenetic event associated with maintenance of super-enhancer stability and stem cell function of PDLSC in mechanoresponse.

## Results

### Transcriptomic profiling reveals global gene expression alterations following mechanical stress

**Fig. 1:**
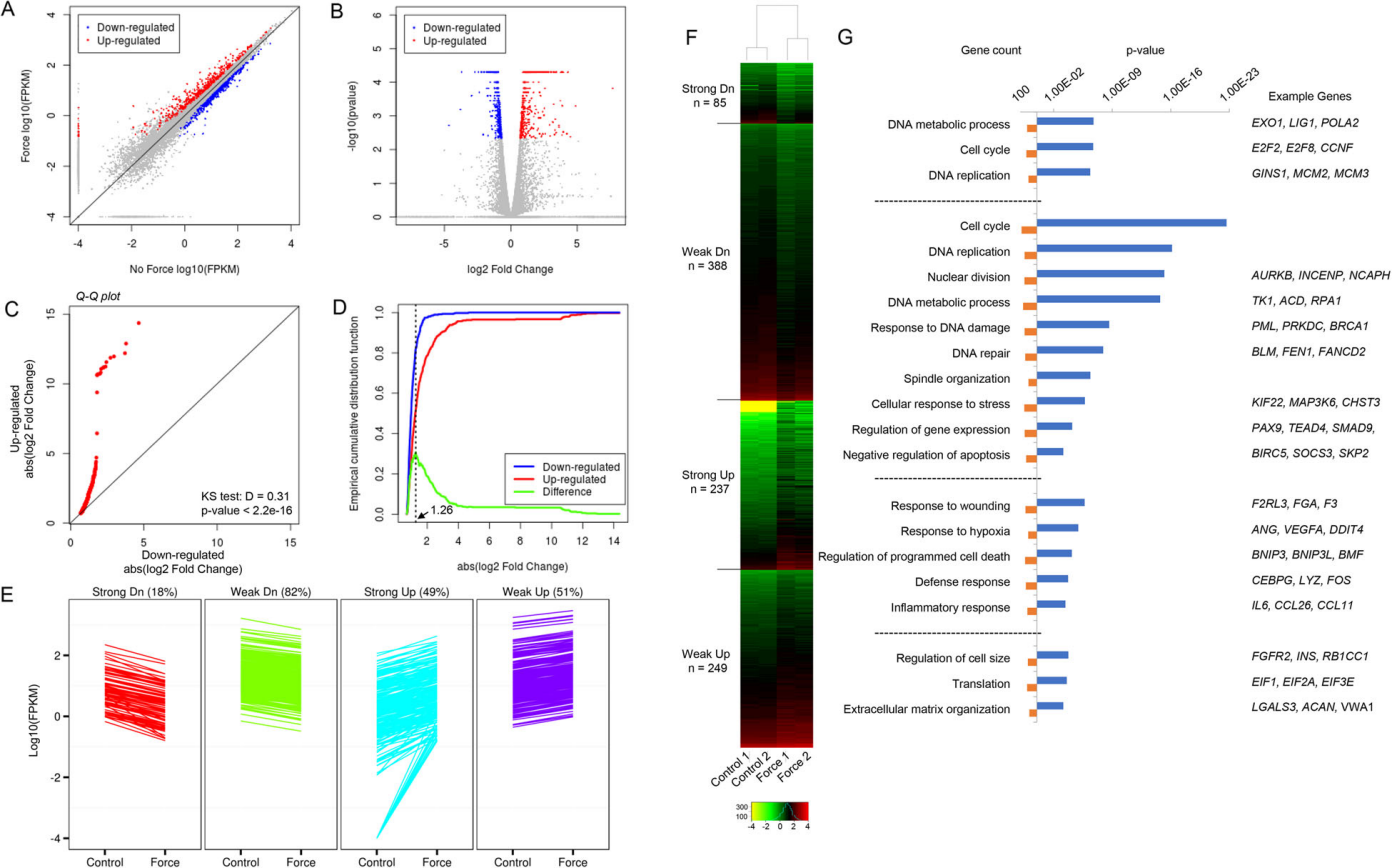
Transcriptomic profiling reveals global gene expression alterations following mechanical stress. **a** PDLSCs were exposed to compressive mechanical force of 1.5 g/cm^2^ for 24 h. The mRNA from two replicates of stressed or control PDLSCs were then collected and subjected to RNA-seq. The data was processed by use of TopHat^77^ and Cufflinks^78^ packages and the mRNA expression levels were shown as log10 transformed FPKM (fragments per kilobase of transcript sequence per millions base). The significantly increased genes were highlighted in red, and the significantly decreased genes were highlighted in blue. **b** Volcano plot demonstrated the significance (log10 transformed) versus fold change of gene expression ratios (log2 transformed). The significantly increased genes were highlighted in red, and the significantly decreased genes were highlighted in blue. **c** Quantile-Quantile (Q–Q) plot demonstrated the differential distribution of absolute expression differences (AED, calculated as absolute value of log2 transformed fold change) for upregulated genes and downregulated genes. KS, Kolmogorov–Smirnov test. **d** Empirical distribution of AED for upregulated and downregulated genes. The difference of these two empirical distribution functions was also plotted. The peak value for the difference curve was indicated. **e** The upregulated and downregulated genes were further divided into four subgroups by their magnitude of force-induced fold changes of gene expression identified in **d**. **f** Heatmap demonstrated the mRNA expression data (log10 transformed FPKM) of genes from the four subgroups identified in **e**. **g** Gene Ontology (GO) analysis for the four subgroups.

During tissue morphogenesis and regeneration, mechanical stress could exert profound and long-term influences on cell identities and properties by acting on a large set of inducible genes^2^. To identify such transcriptomic changes in an unbiased way, we took advantage of high-throughput RNA-sequencing (RNA-seq) to profile the genome-wide mRNA expression patterns following mechanical stress. We used the human dental mesenchymal stem cell purified from periodontal ligament (PDLSC) as our model system, due to their mechanosensitivities and clinical relevance for mechanobiology^20,24^. We exposed these PDLSCs to compressive mechanical force of 1.5 g/cm^2^ for 24 h according to previous reports on PDLSC’s transcriptional responsiveness and viability under these conditions^25–27^. Total mRNA from two replicates of control PDLSCs covered by light glass sheet (0 g/cm^2^) and stressed cells were collected and subjected to RNA-seq. This transcriptional profile analysis revealed a total of ~1000 significantly differentially-expressed genes, among which 486 were upregulated, 473 were downregulated (Fig. 1a). Strikingly, although the total number of genes with increased or decreased expression was comparable, there was a clear distinction on their extent of absolute expression difference (AED) indicated by the specific concentration of downregulated along the diagonal in the scatter plot (Fig. 1a). Volcano plot gave a more lucid demonstration on the closely-neighbored points representing decreasingly expressed genes, in contrast to the widely distributed points for increasingly expressed genes (Fig. 1b). To test whether this trend was statistically significant, we used Quantile-Quantile plot to compare the distribution of absolute expression differences between upregulated genes and downregulated genes, and the result indicated a largely skewed pattern with strong Kolmogorov–Smirnov test significance (Fig. 1c, *p* < 2.2e−16). With this information, we next sought to further distinguish force-induced differentially expressed genes based on their magnitude of expression differences. For this, we plotted the empirical distribution of AED for upregulated and downregulated genes, and used the AED of 1.26 (log2 transformed) which was the peak value for the ecdf (empirical distribution function) difference curve, to define subgroups (Fig. 1d). This criterion finally gave rise to four groups of mechanical force-induced differentially expressed genes, with 85 (18%) strongly downregulated, 388 (82%) weakly downregulated, 237 (49%) strongly upregulated, and 249 (51%) weakly upregulated (Fig. 1e, f).

We next performed Gene Ontology (GO) analysis for these subgroups. A large fraction of downregulated genes was involved in cell cycle control (E2F1, CDCA2, CDC6) and DNA metabolism, such as POLA1, POLE2, PCNA in DNA replication, BRCA1, BLM, FEN1 in DNA damage response and repair (Fig. 1f, g). The majority of strongly induced genes were linked with stress response, ranging from wounding and hypoxia response to inflammation and cell death regulation, while the weakly induced genes were implicated in cell size control and extra cellular matrix organization (Fig. 1g). This disjointed functional annotation between the strongly versus weakly activated subsets of genes implied their mechanoresponses were controlled by distinct regulatory mechanisms (Fig. 1g).

### Gene set enrichment analysis identifies E2F and H3K27me3 signature characterizing force-induced differentially expressed gene groups

**Fig. 2:**
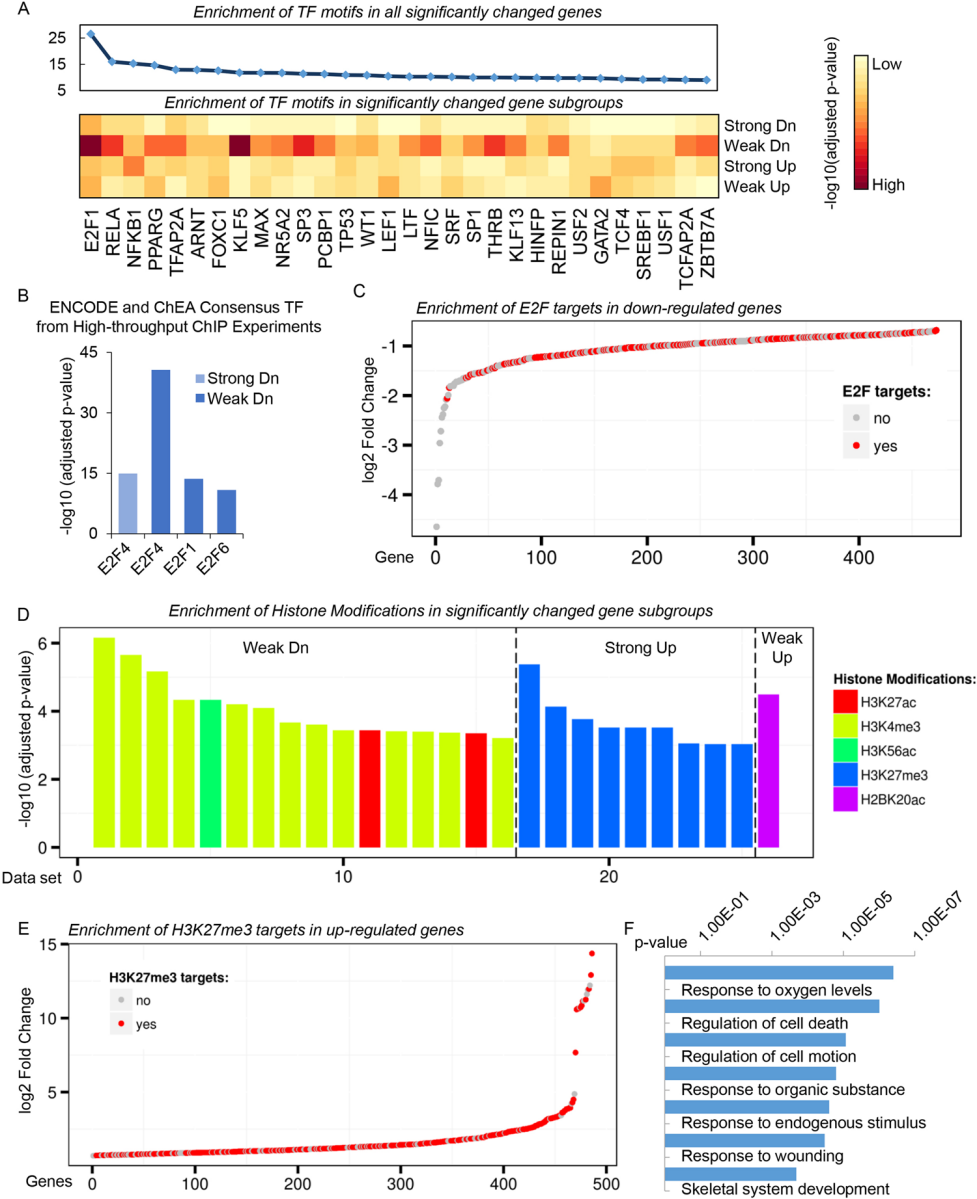
Gene set enrichment analysis identifies E2F and H3K27me3 signature characterizing force-induced differentially expressed gene groups. **a** Enrichr^29^ analysis for transcription factor motif enrichment in the subsets of differentially regulated genes by compressive force identified in Fig. 1. The TF motifs were sorted by their total occurrences in the whole set of differentially regulated genes (upper). Enrichment of TF motifs in any of the four subgroups with significantly changed gene expression was shown as a heatmap (bottom left). The color indicated log10 transformed *p*-value (bottom right). **b** Enrichr analysis for the enrichment of transcription factors by use of actual TF binding profiles from ENCODE^30^. **c** Mechanosensitive genes with significantly decreased expression were sorted by their log2 transformed gene expression differences. E2F targets predicted from **b** were highlighted in red. **d** Enrichr analysis for the enrichment of histone modifications. Each bar indicated a dataset with significant enrichment, and the data sets were sorted by their log10 transformed *p*-values. **e** Mechanosensitive genes with significantly increased expression were sorted by their log2 transformed gene expression differences. H3K27me3 targets predicted from **d** were highlighted in red. **f** Gene Ontology analysis for the H3K27me3-bound subset of upregulated genes.

Given the critical function of known mechanosensitive transcription factors (TF) in transduction and translation of physical signals^28^, we thus asked whether these subsets of differentially regulated genes by compressive force in PDLSCs were enriched with any specific TF targets. For this purpose, we performed gene set enrichment analysis by use of the Enrichr database which integrates a large collection of diverse gene set libraries for the reconstruction of TF network based on their shared targets and binding site proximity^29^. This analysis revealed the enrichment of a panel of TF motifs including E2F1, KLF5, SP3, and RELA in the weakly downregulated genes, while the E2F1 motif was most considerably identified (Fig. 2a). Surprisingly, there was no strong enrichment of any TF motifs among other sets of differentially regulated genes (Fig. 2a). In line with this observation, Enrichr analysis with actual TF binding profiles from ENCODE^30^ suggested a significant occupancy of E2F family TFs including E2F4, E2F1, and E2F6 on the regulatory elements of weakly downregulated genes, while enrichment of E2F4 on the set of strongly downregulated genes could also be marginally detected (Fig. 2b). This point was clearly demonstrated by the prevailing of E2F targets when the downregulated genes were sorted by their expression differences induced by compressive force (Fig. 2c).

Since there were no strong indications for any particular TF that could account for mechanical stress-induced gene activation, we therefore asked whether any histone modifications were specifically enriched in the set with increased expressions. Interestingly, we found a consistent pattern of significant H3K27me3 occupancy on the strongly upregulated gene set across several different data sources, while no other histone modification enrichment could be observed (Fig. 2d, e). Gene Ontology analysis suggested a concordant over-representation of stress response and cell fate determination terms in the H3K27me3-bound subset of upregulated genes (Fig. 2f). In contrast, as the binding sites for E2F family TF are most located near transcription start site^31^, H3K4me3 as an active promoter mark was enriched in the large portion of weakly downregulated genes (Fig. 2d). On the other hand, although H3K27me3 enrichment associated with the weakly upregulated genes did not pass the statistical threshold, a portion of these genes also showed H3K27me3 signature (Fig. 2e).

### Decreased E2F and EZH2 transcription following mechanical stress

**Fig. 3:**
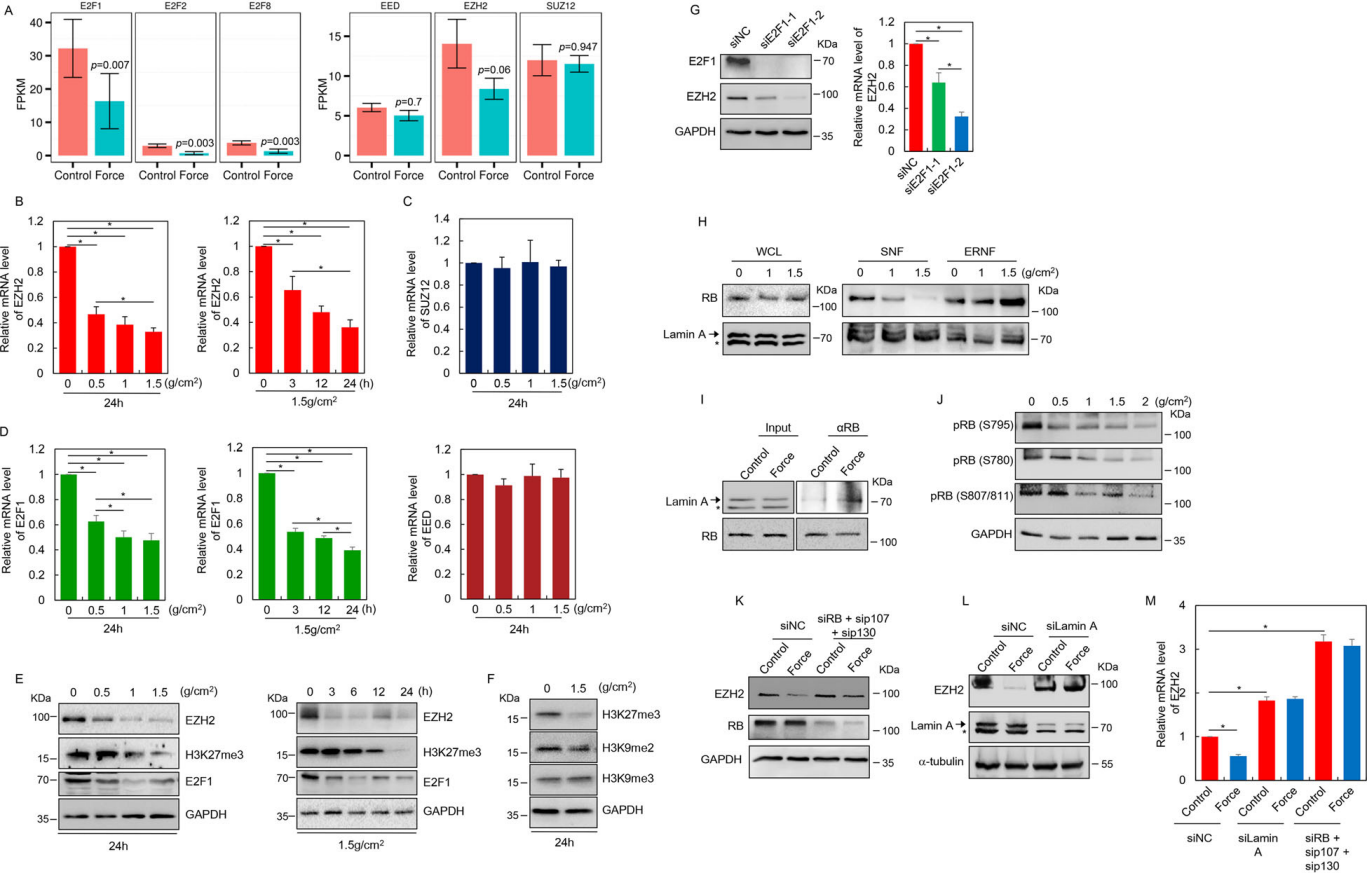
Redistribution of RB from nucleoplasm to lamina accounts for mechanical stress-mediated E2F and EZH2 downregulation. **a** FPKM for indicated genes was shown as barplot. **b**–**e** Expression changes of indicated genes were determined in PDLSCs treated with compressive force of increasing strength for 24 h, or of varying durations at the level of 1.5 g/cm^2^, by RT-qPCR (**b**, **c**, **d**) or western blotting (**e**). **f** Indicated histone modifications in control versus stressed PDLSCs were measured by western blotting analysis. **g** Unstressed PDLSCs were transfected with control or two independent siRNA molecules against E2F1. Western blotting and RT-qPCR analysis were performed to measure the protein and mRNA levels of indicated genes, respectively. **h** The nuclear proteins were separated into extraction-resistant nuclear fraction (ERNF) containing insoluble Lamin A and its associated proteins, and soluble nuclear fraction (SNF) containing other nucleoplasmic proteins, in both control and stressed PDLSCs according to established protocol^35^. These fractions together with the whole cell lysate (WCL) were subjected to western blotting analysis. For the results of Lamin A, the upper band marked with an arrow is Lamin A; the lower band marked with an asterisk is Lamin C. **i** Co-immunoprecipitation assay was performed to examine force-induced interaction changes between RB and Lamin A. **j** PDLSCs were treated with gradually increased compressive force. Phosphorylated RB in these cells was probed using indicated antibodies. **k**–**m** PDLSCs were transfected with control or siRNA against RB family proteins RB, p107 and p130, or Lamin A, and these cells were further exposed to mechanical stress at 1.5 g/cm^2^ for 24 h. Western blotting and RT-qPCR analysis were then performed to examine the expression changes of indicated genes. *p < 0.01; one-way ANOVA (**b**, **c**, **d**, **g**, **m**). Each bar represents mean ± SD for three independent experiments.

Globally altered E2F-target and PRC2-target gene expression promoted us to test whether the transcription of E2F family TFs and PRC2 components were regulated by mechanical stress. Indeed, investigation on the RNA-seq data revealed significantly decreased mRNA levels of E2F1, E2F2, and E2F8 in response to compressive stress (Fig. 3a). Examination of mRNA levels of the three core PRC2 components indicated that the expression of EZH2 rather than SUZ12 or EED was specifically suppressed by physical stress (Fig. 3a). This transcriptional inhibition by mechanical stress was then confirmed through dose-dependent or time-course RT-qPCR analysis. When PDLSCs were treated with gradually increased compressive force, the mRNA level of EZH2 was correspondingly decreased compared to the unaltered SUZ12 or EED expressions (Fig. 3b, c). PDLSCs from seven other donors treated with a compressive force of 1.5 g/cm^2^ for 24 h showed consistent downregulation of EZH2 mRNA levels (Supplementary Fig. 1A). Western blotting analysis validated this downregulation at the protein level (Fig. 3e). We also observed a concomitant erasure of H3K27me3 mark responding to compressive stress in a dose-dependent manner (Fig. 3e). In support of the specially decreased EZH2 expression, both H3K9me2 and H3K9me3 as marks for heterochromatin were left unchanged, implying that physical strain associated chromatin remodeling did not uniformly occur on repressive epigenetic domains (Fig. 3f). Time-course RT-qPCR and western blotting analysis suggested that mechanical force-mediated transcriptional inhibition of EZH2 was progressively taking place, and the global H3K27me3 level showed a proportional decrease (Fig. 3b, e). Examination of E2F1 expression changes revealed a similar dose-dependent and time-dependent transcriptional reduction induced by compressive stress (Fig. 3d, 3e). Moreover, this compressive force invoked Ezh2 downregulation could also be observed in mouse orthodontic tooth movement (OTM) model—a more physiologically relevant context (Supplementary Fig. 1B).

### Redistribution of RB from nucleoplasm to lamina accounts for mechanical stress-mediated E2F and EZH2 downregulation

We next sought to determine the molecular mechanisms for mechanical force-mediated suppression of E2F and EZH2 expressions in PDLSC. Early studies have established EZH2 as a direct transcriptional target of E2F family TFs for cancer cell proliferation^32^, we thus tested whether this regulation was preserved in the non-transformed PDLSCs. Since E2F1 is the major E2F TF in our PDLSC model (Fig. 3a), we transfected two independent siRNA molecules against E2F1 in the unstressed PDLSCs. As expected, western blotting and RT-qPCR analysis indicated that the protein and mRNA levels of EZH2 were severely suppressed following E2F1 removal (Fig. 3g).

Among all the potential regulators connecting E2F/EZH2 and mechanosignaling, retinoblastoma protein (RB) is particularly interesting. This is because that RB could not only function as a major E2F regulator by physically interacting with E2F to mask its transactivation domain^33^, but it can also be tethered to lamina^34^, thus holding a potential linkage with nuclear skeleton-mediated mechanosignaling. To test whether RB shows mechanosensitivities with physical stress-regulated dynamic association with lamin, we performed biochemical fractionation^35^ to isolate insoluble Lamin A and its associated proteins within nuclear envelope from the extraction-resistant nuclear fraction (ERNF), and other nucleoplasmic proteins from the soluble nuclear fraction (SNF). In the unstressed PDLSCs, comparable levels of RB were found in ERNF and SNF (Fig. 3h). However, application of compressive force promoted its redistribution from SNF to ERNF; and notably, when the PDLSCs were exposed to 1.5 g/cm^2^ stress, RB showed a nearly complete relocalization to ERNF (Fig. 3h), suggesting that mechanical stress could induce RB enrichment on nuclear skeleton. Since the expression level and distribution of Lamin A were nearly unchanged (Fig. 3h), increased association between RB and lamin should account for this relocalization (Fig. 3i and Supplementary Fig. 2). In parallel to the enrichment of RB on lamina, RB phosphorylation was substantially decreased by compressive stress (Fig. 3j), indicating a formation of the inactive RB-E2F complex amenable to active repression of downstream gene transcription.

Given the autoregulatory control of E2F1 expression by RB-E2F complex^36^ and E2F-dependent EZH2 expression in PDLSCs (Fig. 3g), it is interesting to ask whether mechanical stress elicited transcriptional suppression of E2F1 and EZH2 was mediated by RB. To test this, PDLSCs were co-transfected with siRNA molecules against RB, p107 and p130—two retinoblastoma protein family members showing functional redundancy with RB^37^—followed by exposure to mechanical stress. Western blotting and RT-qPCR analysis suggested that force-induced EZH2 downregulation was nearly abolished upon their co-depletion (Fig. 3k, m). Importantly, nuclear envelope defects with Lamin A depletion led to unreduced transcription of EZH2 in response to mechanical stress as well, hence pointing to the significance of lamin-dependent RB tethering for mechanosignaling in PDLSCs (Fig. 3l, m).

### Epigenomic analysis reveals force-induced dynamics of H3K27me3 landscape surrounding the differentially regulated mechanoresponsive genes

**Fig. 4:**
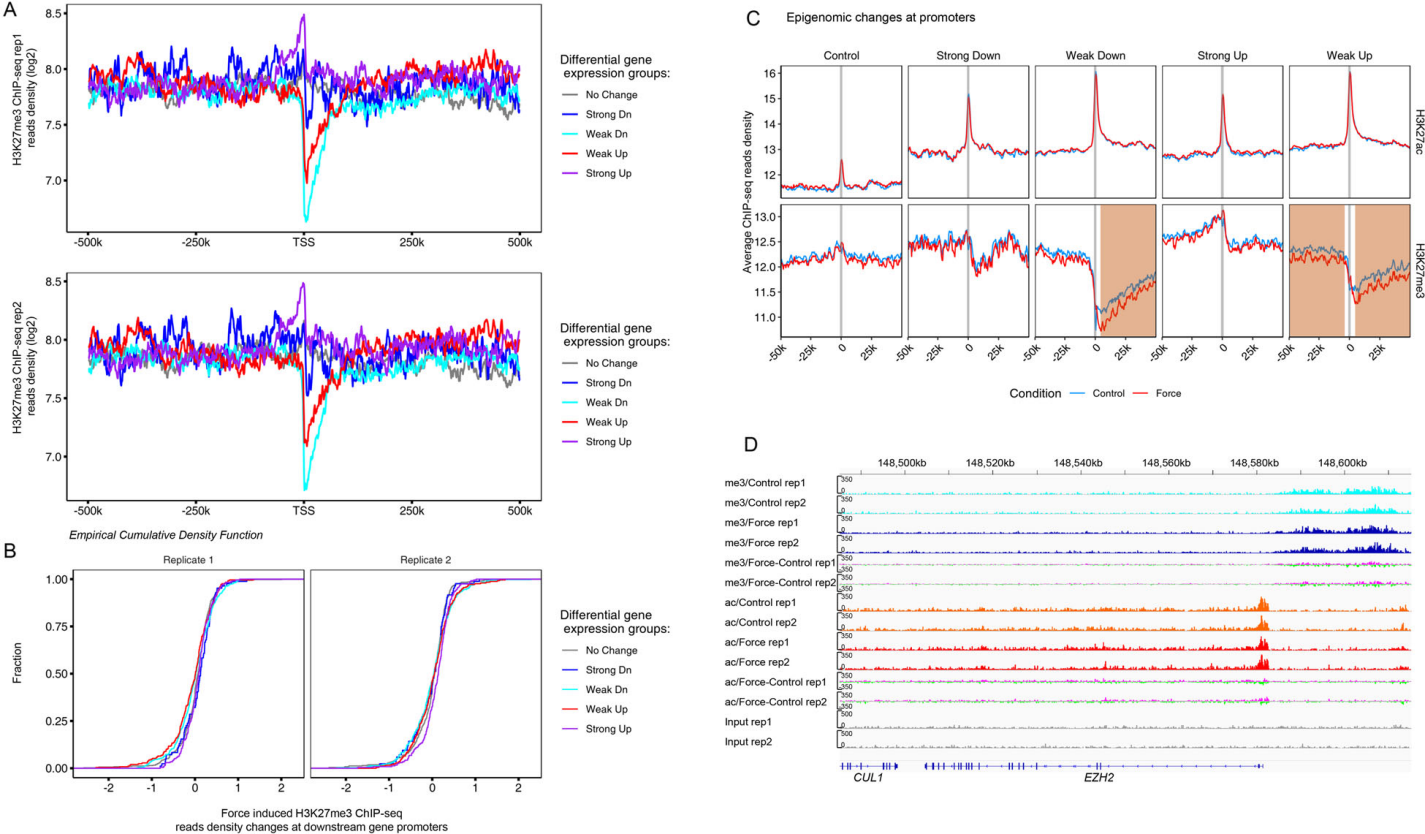
Epigenomic analysis reveals force-induced dynamics of H3K27me3 landscape surrounding the differentially regulated mechanoresponsive genes. **a** Chromatin immunoprecipitation coupled with high-throughput sequencing (ChIP-seq) using antibodies against H3K27me3 was performed in unstressed PDLSCs with two biological replicates. Aggregate plot for each of the four differentially regulated groups in addition to a control group with no significant expression change showed average H3K27me3 occupancy in a 1 million base window centered by transcription start sites (TSS) of downstream genes. rep, replicate. **b** ChIP-seq analysis using antibodies against H3K27me3 was also performed in the stressed PDLSCs. Empirical cumulative distribution function for the mechanical stress induced H3K27me3 binding differences within the central window enclosing TSS of the indicated differentially regulated sets of genes was plotted. **c** Aggregate plot showed the average H3K27ac and H3K27me3 occupancy along the 100 kb window centered by TSS of downstream genes from each of the five groups defined in **a** in the absence or presence of mechanical stress. Regions with detectable H3K27me3 occupancy changes were shaded. **d** ChIP-seq profiles along the EZH2 locus for input or the indicated histone modifications.

To understand the consequences of EZH2 reduction in mechanoreponses, we firstly profiled the genome-wide H3K27me3 occupancy in the resting PDLSCs by chromatin immunoprecipitation coupled with high-throughput sequencing (ChIP-seq). Since the H3K27me3 mark is broadly extended into large domain^38^, we investigated its enrichment pattern in a million base window centered by transcription start sites (TSS) of downstream genes. Aggregate plot for the four differentially regulated groups in addition to a control set of genes with no significant expression changes revealed a robust enrichment of H3K27me3 surrounding the strongly induced genes, which was peaked near the TSS and further spreading ~50 kb upstream (Fig. 4a). In sharp contrast to this, both the weakly downregulated and upregulated genes showed a prominent depletion of H3K27me3-containing nucleosomes downstream the TSS, implying distinctive regulatory mechanisms underlying their response to mechanical stress (Fig. 4a).

The computational prediction using epigenomic annotation (Fig. 2) and the global decline of H3K27me3 levels following compressive stress (Fig. 3) suggested a genome-wide remodeling of H3K27me3 landscape upon physical stress. In order to probe this dynamic, we performed ChIP-seq experiment in the stressed PDLSCs and further compared their H3K27me3 binding profile with the control cells. Empirical cumulative distribution function of the H3K27me3 binding differences within the central window enclosing TSS, however, indicated negligible changes induced by mechanical stress for any differentially regulated sets of genes compared to the unchanged background gene set (Fig. 4b). Aggregate plot for the average H3K27me3 enrichment at a larger window of 100 kb, however, unmasked force-induced H3K27me3 changes, where the two groups with either weakly upregulated or weakly downregulated transcription showed decreased H3K27me3 occupancy following compressive force stress, compared to the strongly upregulated and downregulated groups and the control group without mechanosensitivities (Fig. 4c). Furthermore, this decreasing trend was prominent both at upstream and downstream regions with respect to the TSS of weakly increased genes, whereas it was much stronger at downstream compared to upstream of the TSS of weakly decreased genes, correlating with the possible de-repression of H3K27me-bound upstream *cis*-regulatory elements of weakly activated genes in response to mechanical stress (Fig. 4c).

The promoter of weakly regulated genes showed substantially low abundance of H3K27me3-containing nucleosomes in unstressed cells (Fig. 4a, c), in sharp contrast to the group of strongly upregulated genes, whose promoters were enriched with H3K27me3 at basal state but were devoid of expected H3K27me3 loss following force application despite their de-repressed transcription (Fig. 4a, c). This lack of correlation suggested that EZH2-associated H3K27me3 deposition might be imparted with structural regulatory function of epigenome in mechanoresponse, rather than be directly implicated in force-induced transcriptional activation or suppression. In agreement with this notion, profiling of H3K27ac, an active promoter and enhancer mark^18^, revealed no significant changes at promoters of any of the five differentially regulated gene groups. Moreover, the two weakly regulated gene groups showed greater enrichment of H3K27ac both in the control and stressed cells compared to other groups, concordant with their higher levels of basal transcription (Fig. 1f).

On the other hand, a specific inspection of H3K27me3 and H3K27ac dynamics at the *EZH2* locus, demonstrated that although global mRNA and protein levels of EZH2 decreased in response to mechanical stress, EZH2 promoter showed substantially increased H3K27me3 enrichment along a ~20 kb H3K27me3-enriched domain upstream of the TSS, implying an auto-inhibitory loop to suppress EZH2 transcription in mechanoresponse, in line with RB-dependent and PRC2-dependent H3K27me3 deposition model for gene silencing (Fig. 3)^39^.

### Flattening super-enhancers show increased H3K27ac entropy along with preserved EZH2 expression in response to mechanical stress

**Fig. 5:**
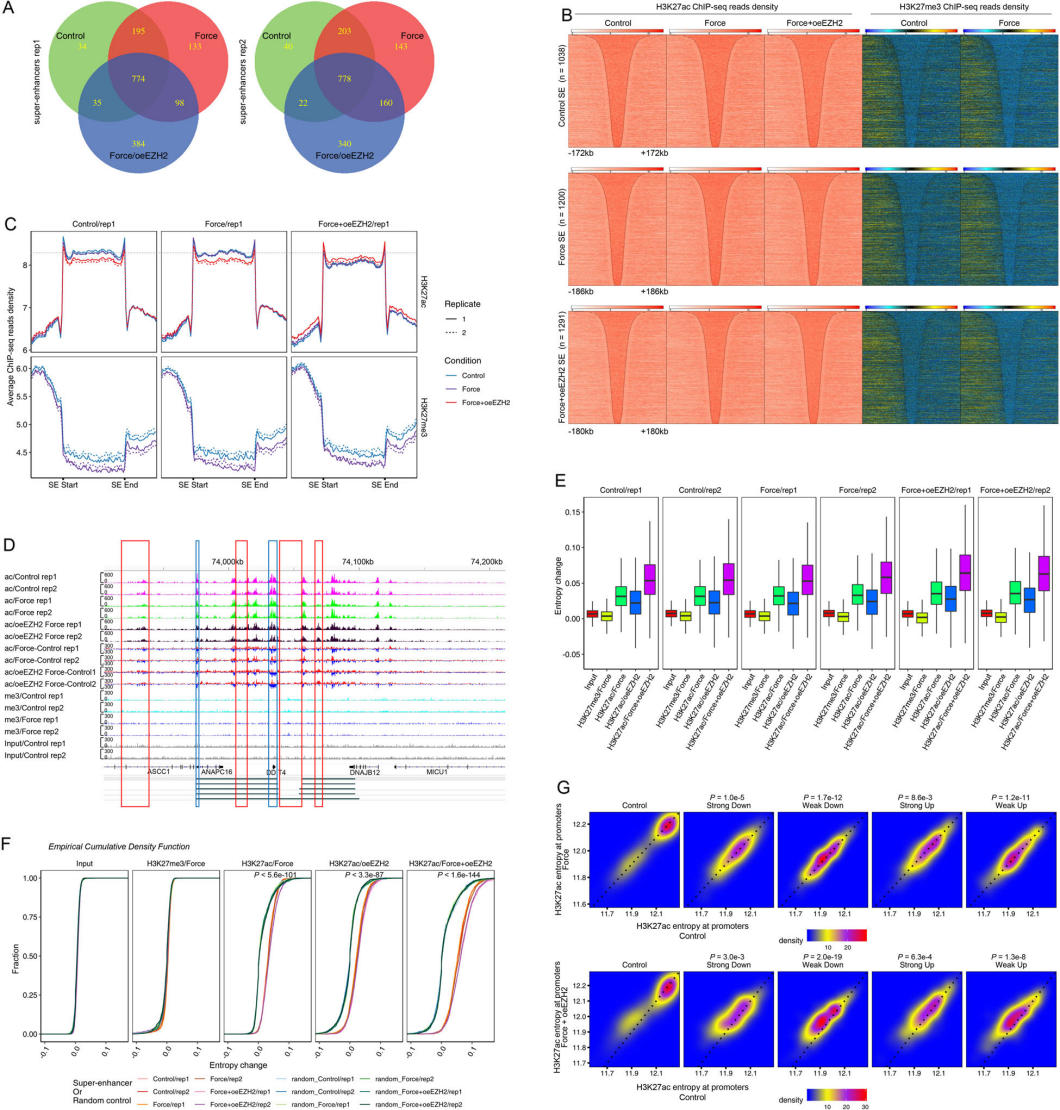
Flattening super-enhancers show increased H3K27ac entropy along with preserved EZH2 expression in response to mechanical stress. **a** We prepared adenoviral EZH2 for transient expression to compensate force-induced EZH2 reduction (oeEZH2) and also fine-tuned the amount of infected virus to keep the total EZH2 levels in mechanical stressed PDLSCs comparable to unstressed cells. The H3K27ac landscapes in control PDLSCs in the absence or presence of mechanical stress and H3K27ac landscape in the stressed oeEZH2-PDLSCs were determined in two biological replicates. Super-enhancers (SEs) were called by use of ROSE algorithm^17^ with default parameters for each H3K27ac ChIP-seq library. Pie chart plot demonstrated the number of unique and overlapped SEs with respect to the indicated conditions. **b** ChIP-seq reads density (log2 transformed) for H3K27ac and H3K27me3 in each of the indicated conditions (specified on the top) was plotted in heatmap. Each row represented the same SE called from the indicated PDLSC (replicate 1, specified on the left). SEs were sorted by their length and aligned on the center. The SEs with stronger H3K27me3 enrichment on the right side were flipped horizontally. **c** SEs identified from each replicate of PDLSCs under the indicated conditions were arranged as in **b** and were divided into same number of bins. Aggregate plot showed the average H3K27ac and H3K27me3 occupancy along each bin within SEs. ChIP-seq libraries used for this plot were indicated on the top. **d** ChIP-seq profiles along the indicated locus for input or the indicated histone modifications. Regions with loss of strong H3K27ac peaks following the compensation of deceased EZH2 expression in mechanoresponse were boxed in blue. Regions with gain of continuous but small H3K27ac hillocks were boxed in red. **e** Epigenetic entropy of a SE was defined as expected information content with respect to the probability mass function calculated as normalized reads density along that SE. Boxplot demonstrated the distribution (showing the five summary statistics) of entropy differences or changes with respect to the group of SEs identified from the indicated PDLSCs (specified on the top). Background entropy differences were calculated with the use of two input libraries. Entropy changes for H3K27me3 or H3K27ac when examining the effect of force, oeEZH2, or their combination were indicated below. **f** Empirical cumulative density function plot for the background entropy differences or the epigenetic entropy changes as in **e** with respect to the group of SEs identified from the indicated PDLSCs or a control set of random background regions with similar length distribution as SEs under each condition. Wilcoxon signed-rank test was used to determine the statistical significance. The greatest *p*-value was indicated when comparing epigenetic entropy differences or changes with respect to each group of SEs and their relevant control intervals. **g** Scatter plot comparing the H3K27ac entropy differences under the indicated conditions with respect to the promoters of force-induced differentially regulated group of genes. Wilcoxon signed-rank test was used to determine the statistical significance.

With the finding on force-induced decreased enrichment of H3K27me3 at promoters of weakly regulated genes, which also showed high level of H3K27ac occupancy indicative of active chromatin context, we next sought to extend this observation by determining the epigenomic states of super-enhancer in mechanoresponse, which are enhancer clusters with unusually high levels of H3K27ac occupancy and enhancer activity, and critically control the expression of cell identity-associated genes^16,17^. We thus profiled the H3K27ac landscapes in control PDLSCs in the absence or presence of mechanical stress in two replicates (Figs. 4c and 5). To unveil the specific contribution of EZH2 downregulation in force-elicited epigenetic dynamics, we prepared adenoviral EZH2 for transient expression to compensate force-induced EZH2 reduction and to avoid uncontrolled long-term effect with the use of retroviral or lentiviral system, and also fine-tuned the amount of infected virus to keep the total EZH2 levels in mechanical stressed PDLSCs comparable to unstressed cells (Supplementary Fig. 3A). We then profiled the H3K27ac landscapes in these cells in the presence of mechanical stress, and called super-enhancers using ROSE^17^ with same parameters from each H3K27ac ChIP-seq library. Overlap analysis for these SEs indicated that simple compressive force exposure elicited a relatively marginal SE diversification, whereas the interference of EZH2 decline led to a larger set of unique SEs in the presence of mechanical stress compared to the unstressed or stressed control cells, suggesting preserved EZH2 expression could affect SE composition in force-induced epigenomic remodeling (Fig. 5a). Importantly, heatmap and aggregate plots showing both H3K27ac and H327me3 reads distribution within the SEs identified under each condition revealed a common theme of H3K27ac clustering in SEs: SEs were tightly bounded by H3K27me3 domain at one side regardless of mechanical stress (Fig. 5b, c). This theme was widespreading and these observations were reproducible (Supplementary Fig. 3B, C).

The packing of H3K27ac-enriched active domain and H3K27me3-enriched repressive domain at the SE boundary implied a functional antagonism between PRC2 and P300 to deposit these two marks, which further raised the possibility that aberrant EZH2 retention in mechanoresponse would promote the encroachment of SE by PRC2-catalyzed H3K27me3 modification. Indeed, we observed a significant decrease of H3K27me3 occupancy within the SE and neighboring chromatin at the side showing lower level of H3K27me3 in response to mechanical stress in control cells, complying to force-induced EZH2 reduction (Fig. 5c and Supplementary Fig. 3C). This reduction of H3K27me3 deposition was accompanied by SE stabilization as indicated by the maintenance of H3K27ac levels within SEs in the mechanoresponse of control cells (Fig. 5c and Supplementary Fig. 3C). In agreement with the disruptive roles of EZH2 retention for SE function, enforced EZH2 preservation during mechanical stress led to a grossly decreased H3K27ac enrichment at SEs (Fig. 5c and Supplementary Fig. 3C). Moreover, analysis of H3K27ac occupancy against the SEs identified from EZH2-preserved stressed PDLSCs suggested that this set of SEs, less enriched with H3K27ac, might be functionally less competent than SEs from control cells (Fig. 5c and Supplementary Fig. 3C).

In addition to the decreasing trend of aggregate SE H3K27ac enrichment following the compensation of deceased EZH2 expression, inspection of individual sites identified both loss of strong H3K27ac peaks and gain of continuous but small H3K27ac hillocks in the trough separating original peaks within SEs (Fig. 5d and Supplementary Fig. 3D). To quantify the overall SE functionality by prioritizing sharp H3K27ac peaks and negating small H3K27ac hillocks, we borrowed the concept of Shannon entropy and measured epigenetic entropy of a SE as expected information content with respect to the probability mass function calculated as normalized reads density along that SE. Hence the SE with decreased H3K27ac entropy should have roughly increased amount of sharp peaks, which we also termed as super-enhancer polarization. Examination of force-induced epigenetic entropy changes of SEs identified from each condition suggested that compared to the nearly unchanged entropy with respect to input library, H3K27me3 also showed negligible entropy changes following mechanical stress, while the H3K27ac entropy was greatly increased in mechanoresponse across each SE set (Fig. 5e). Importantly, enforced EZH2 retention showed an additional effect, which led to the mostly significant HK27ac entropy increase induced by force (Fig. 5e). Using a control set of random background regions with similar length distribution as SEs identified in each condition, empirical cumulative density function plot for force-induced epigenetic entropy changes also demonstrated the mostly significant increase of H3K27ac entropy in EZH2 preserved PDLSCs (Fig. 5f). Interestingly, in line with these changes in SE, scatter plot comparing the H3K27ac entropy differences with respect to the promoters of force-induced differentially regulated genes demonstrated the most significant gain of H3K27ac entropy associated with compensation of deceased EZH2 expression after exposure to compressive force took place in the weakly upregulated and downregulated gene groups (Fig. 5g). Together, these data suggested that the interference of force-induced EZH2 downregulation could negatively affect epigenome stability through attenuating H3K27ac deposition and super-enhancer polarization in mechanoresponse.

### Spatial Relationship between EZH2 and H3K27ac in mechanoresponse

**Fig. 6:**
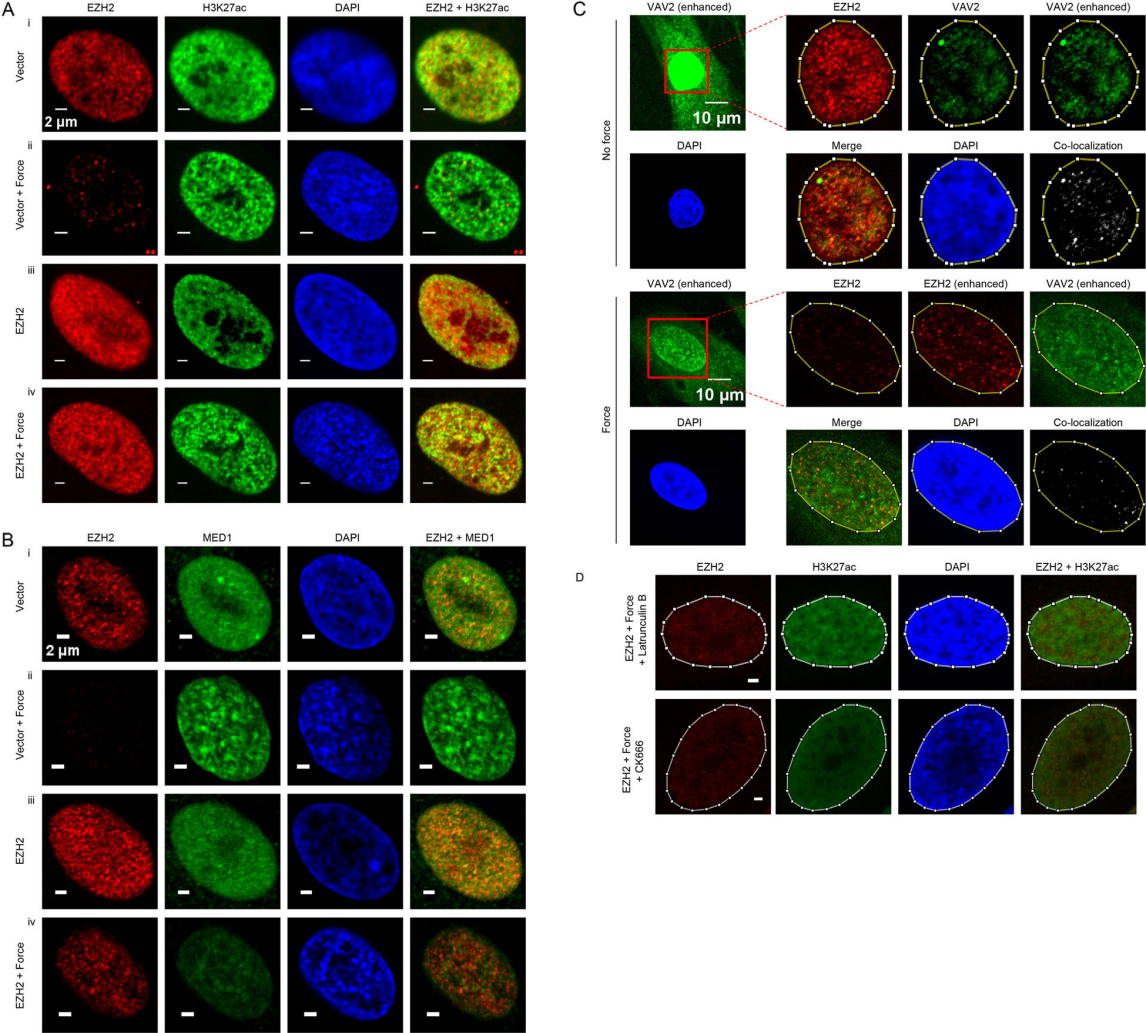
Spatial Relationship between EZH2 and H3K27ac in mechanoresponse. **a** PDLSCs were infected with control or adenoviral EZH2 to compensate force-induced EZH2 reduction. Immunofluorescence imaging of EZH2 and H3K27ac was then performed in these cells in the absence or presence of compressive force. The scale bar indicated 2 µm. **b** PDLSCs were treated as in **a** for the immunofluorescence imaging of EZH2 and MED1. The scale bar indicated 2 µm. **c** Immunofluorescence imaging of EZH2 and VAV2 was performed in unstressed control and compressive force stressed PDLSCs. Image processing was same as in **a**, except for the enhanced signal presentation with improved contrast that has been indicated. Co-localization analysis was done with use of ImageJ program^83^. **d** Compressive force stressed PDLSCs were treated with actin filament polymerization inhibitor Latrunculin B or ARP2/3 inhibitor CK666 before immunofluorescence imaging analysis of EZH2 and H3K27ac. The scale bar indicated 2 µm.

The overall decreased H3K27ac occupancy and increased H3K27ac entropy at stressed SEs driven by compensation of declined EZH2 expression might reflect an elevation of PRC2 targeting to enhancers and SEs associated with excessive EZH2 levels in the presence of mechanical stress. To address this point, we performed immunofluorescence experiment with antibodies against EZH2 and H3K27ac to measure the correlation between their spatial distribution. The results indicated that in control cells, EZH2 proteins were condensed in foci and H3K27ac signals were distributed in large domains across the whole nucleus (Fig. 6ai). Interestingly, application of compressive force resulted in decreased total EZH2 protein levels and a smaller number of EZH2 condensates (Fig. 6aii). In the mechanically stressed PDLSCs infected with same dose of EZH2-expressing adenovirus as above to recompense reduced EZH2 expression (Supplementary Fig. 3A), increased numbers of nuclear EZH2 puncta were observed (Fig. 6aiv). Importantly, force-induced increase of spatial overlapping between dense H3K27ac clumps and EZH2 puncta could also be detected, suggesting improved co-localization between ectopically preserved EZH2 and H3K27ac-decorated chromatin in mechanoresponse (Fig. 6aiv). On the other hand, single EZH2 overexpression in the absence of mechanical stress was not sufficient to drive the entanglement of EZH2 foci and H3K27ac condensate to the level when force was further applied, supporting an essential role of mechanotransduction in promoting EZH2 targeting to H3K27ac-charaterized regions (Fig. 6aiii).

Because of the strong overlap of fluorescence signals between puncta of mediator component MED1 and labeled super-enhancer DNA in vivo^40^, we further examined the dynamics of MED1 as SE proxy following mechanical stress with respect to EZH2 levels (Fig. 6b). The results indicated that in unstressed PDLSCs, a fraction of MED1 proteins were aggregated into discernible nuclear bodies (Fig. 6bi), concordant with its roles in binding of the co-operative and multivalent super-enhancers^19^. This condensation pattern of MED1 was not reduced but even improved after exposure to mechanical stress, along with the greatly decreased EZH2 signals (Fig. 6bii). Importantly, in line with the western blotting results showing overall decrease of nuclear MED1 levels upon compensation of force-induced EZH2 downregulation in stressed PDLSCs (Supplementary Fig. 4A), a representative image demonstrated attenuation of MED1 fluorescence and foci density (Fig. 6biv), supporting the decreased SE stabilization by interference of EZH2 reduction in nuclear mechanotransduction.

Interestingly, cytoplasmic EZH2 has been demonstrated to be physically and functionally associated with VAV1, a member of VAV family guanosine nucleotide exchange factors (GEFs) catalyzing the exchange of guanosine diphosphate (GDP) to guanosine triphosphate (GTP) for Rho GTPases activation^41^. Owing to the key roles of Rho GTPases in cytoskeleton remodeling, organelle transport and movement^42^, it is intriguing to test whether the VAV family GEFs are implicated in mechanical stress-induced nuclear EZH2 dynamics. Querying the mRNA expression profiles of PDLSCs, we found a specific and high expression of VAV2 but no other VAV members in PDLSCs (Supplemental Fig. 4B). We thus performed immunofluorescence analysis with VAV2 antibody, and the results indicated that along with detectable VAV2 signals diffused in cytoplasm, a significant portion of VAV2 proteins were present in nucleus in the absence of mechanical stress (Fig. 6ci). Co-localization analysis demonstrated a fraction of EZH2 foci aligned with nuclear VAV2 puncta (Fig. 6ci), supporting a conserved association theme for EZH2 and VAV factors in nucleus. Although compressive force application caused a reduction of nuclear VAV2 signals relative to its cytoplasmic distribution in the company of decreased EZH2 foci formation, co-localization between VAV2 and EZH2 puncta could still be observed (Fig. 6cii), implying a preservation of EZH2-VAV2 interaction with respect to the retained EZH2 proteins.

We then further asked whether the actin filament network acting downstream of VAV GEFs participated in force-induced spatial changes of EZH2 and H3K27ac marked enhancers. Immunofluorescence results in PDLSCs with aberrant EZH2 retention indicated that inhibition of actin filament polymerization by Latrunculin B greatly suppressed the formation of nuclear EZH2 foci, H3K27ac condensates and their spatial overlap (Fig. 6di). Meanwhile, treatment of ARP2/3 inhibitor CK666 to block ARP2/3 associated actin nucleation led to similar effects in EZH2 compensated and stressed PDLSCs (Fig. 6dii). These results together suggested that compressive force activated F-actin and ARP2/3 might be involved in VAV2-transduced and actin filament-dependent movement of excessive EZH2, whereas the increased co-localization between PRC2 and H3K27ac-enriched SE could cause a shift of local balance between PRC2 and its antagonizing acetyltransferase that deposits H3K27ac.

### PDLSCs with preserved EZH2 expression are deficient for induced differentiation after exposure to compressive force

**Fig. 7:**
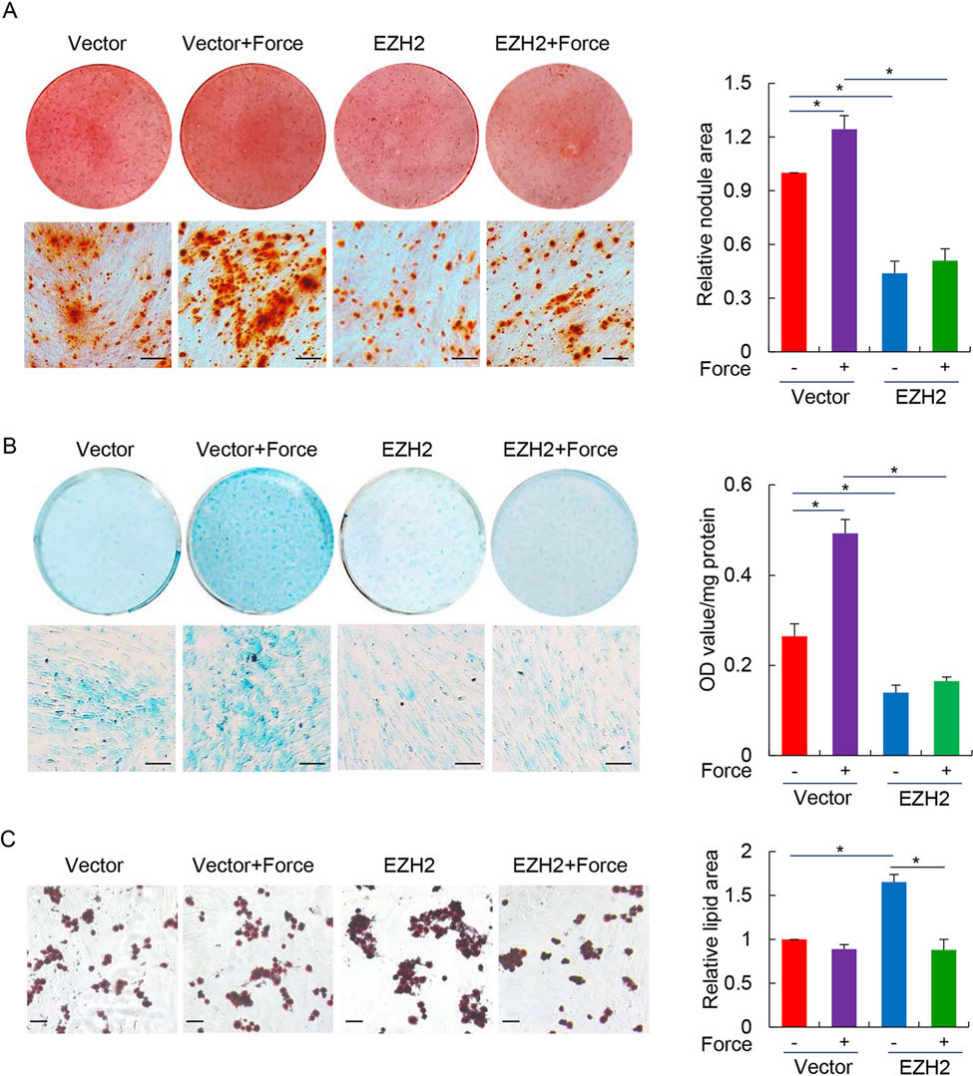
PDLSCs with preserved EZH2 expression are deficient for induced differentiation after exposure to compressive force. **a** PDLSCs infected with adenoviral EZH2 or the control vector were exposed to compressive mechanical force at 1.5 g/cm^2^ for 24 h. Then the cells were cultured in osteogenic differentiation medium for 3 weeks before subjected to Alizarin Red (AR) staining. The representative images were shown on the left. The scale bar indicated 500 µm. Comparison of total areas of mineralized nodules in different groups was shown on the right. **b** PDLSCs were subjected virus infection and mechanical stress treatment as in **a**, and then cultured in chondrogenic differentiation medium for 21 days before Alcian Blue (AB) staining. The representative images were shown on the left. The scale bar indicated 500 µm. The result of quantitative analysis was shown on the right. **c** PDLSCs were subjected virus infection and mechanical stress treatment as in **a**, and then cultured in adipocytic differentiation medium for 21 days before Oil Red O (ORO) staining. The representative images were shown on the left. The scale bar indicated 250 µm. The comparison of the total areas of lipid droplets in different groups was shown on the right. *p < 0.01; one-way ANOVA. Each bar represents mean ± SD for three independent experiments.

Since a large cohort of super-enhancer controlled genes are involved in the maintenance of cell identity and memory^16^, it is interesting to ask whether aberrant EZH2 retention associated epigenomic alterations within SE would impact the stem cell function of PDLSC. Among the multiple choices for lineage commitment of PDLSC as a special type of mesenchymal stem cell, differentiation of PDLSC into osteoblast, chondrocyte, and adipocyte has potential values for clinical application and has been extensively studied^43,44^. Therefore, we prepared control and EZH2-compensated PDLSCs and exposed them to compressive force of 1.5 g/cm^2^ for 24 h, and then cultured these or unstressed PDLSCs in osteogenic, chondrogenic, or adipogenic medium to induce their differentiation. We then performed Alizarin Red (AR) staining, Alcian Blue (AB) staining and Oil Red O (ORO) staining assays to measure the efficiency of PDLSC commitment into osteoblast, chondrocyte, or adipocyte, respectively. Consistent with the mechanosensitivities of PDLSC^45,46^, compressive force exposure promoted the osteogenic and chondrogenic differentiation, but suppressed the adipogenic differentiation of PDLSC, indicated by increased mineral nodule areas and strengthened AB staining, in contrast to the decreased lipid formation as shown by ORO staining (Fig. 7a–c). In line with the established role of EZH2 in adipogenesis^47^, we found single EZH2 overexpression promoted adipogenic differentiation of PDLSCs (Fig. 7c), but greatly suppressed their commitment into osteogenic or chondrogenic lineages, indicating a reciprocal control of PDLSC differentiation into osteoblast/chondrocyte and adipocyte^22^. Importantly, consistent with the attenuated H3K27ac deposition and polarization of super-enhancer in mechanoresponse, interference of force-induced EZH2 downregulation suppressed differentiation of PDLSC into all of the three lineages after exposure to mechanical stress, implying a fundamental deficiency rather than defects of specific pathway associated with aberrant EZH2 preservation (Fig. 7a–c). Furthermore, comparison of the expression changes of cementoblastic differentiation marker genes CEMP-1 and CAP induced by compressive stress in control versus EZH2 ectopically preserved PDLSCs indicated that force treatment promoted the expression levels of both CEMP-1 and CAP in control cells; however, EZH2 overexpression suppressed this trend (Supplementary Fig. 5), implying the functional impact of force-induced EZH2 downregulation in cementoblastic differentiation.

## Discussion

Increasing body of evidence suggests that mechanical stresses, like other developmental cues, act on chromatin structure to shape cell’s adaption for physical niche and tissue geometry^11,48^. These actions showed a “mechanical memory” pattern^11,48^, and the sequence-specific mechanosensitive transcription factors such as YAP/TAZ and RUNX2 were demonstrated to be the core memory elements by storing information from past physical environments and contributing to lineage choice^11^. However, in this study, instead of identifying known mechanosignaling pathways controlled by specialized factors^2^ in dental mesenchymal stem cell’s response to compressive force, we reported that generic and fundamental programs, including stress response, cell cycle control and cell fate determination were significantly altered in mechanical stress mediated transcriptomic dynamics. Through a series of epigenomic and molecular biology approaches, we delineated the contribution of E2F and EZH2 as the master regulators for these changes. This finding complies with the well-recognized epigenetic function of PRC2 complex in cell identity maintenance in both pluripotent and differentiated states^12,15^.

Although the unequivocally precise mechanisms underlying the requirement of cell cycle exit for terminal differentiation is lacking, association between cell cycle progression and cell fate specification has been frequently observed^49^. This phenomenon is partially explained by the activation of differentiation genes in G1 phase and the instability of pluripotency network in stem cells with elongated G1 when confronting with a variety of pro-differentiation signals^50^. For the PDLSC, we identified declined expression of E2F family TFs and reduced RB phosphorylation, which could contribute to mechanical cues-mediated postponement of G1/S transition and hence give more opportunities for PDLSC to make cell fate decision. Globally decreased EZH2 expression would add another layer for this regulation, by imposing an imbalance for H3K4me3 and H3K27me3 marks on bivalent domains within expanded G1 phase^51^ to increase chromatin plasticity. The two major constituents accounting for force-elicited transcriptomic changes in PDLSCs are mechanistically linked with respect to their transcriptional regulations by the retinoblastoma tumor suppressor gene. These regulatory circuits include the RB/E2F/cyclin positive-feedback loop^52^ and RB/E2F mediated transcriptional repression of EZH2^32,39^. Indeed, conditional Rb knockout mice with deregulated E2F activities and impaired cell cycle exit showed abnormal osteoblast differentiation and bone formation^53^. Importantly, the p107/p130-double-null mice showed a clear developmental delay in tooth germs^54^. In these Rb-deficient mice, incisor microdontia with hypoplasia of the odontoblast layer and cell disorganization could be commonly observed. Even a few instances showed anodontia and an extreme case lacked the incisors completely. Mechanical cues mediated RB communication with nuclear envelope would bypass the mechanotransduction initialized at the cell membrane, whereas the integrin-based adhesion complexes as the mechanosensors showed extensive connections with phosphorylation dependent biochemical pathways. Differences in these two routes are highlighted by nuclear lamina associated profound epigenomic remodeling compared to the specifically activated transcription factor networks by cytoskeleton^55^; and obviously, their entanglement warrants further investigations.

Since the interphase nucleus is much stiffer than cytoplasm, and both the lamina and chromatin contribute to its stiffness and mechanics^56^, it is not surprising that chromatin based epigenetic regulation for gene transcription is integrated into mechanotransduction signaling^2,57^. For example, fluid shear stress could activate histone deacetylase 3, leading to p53 deacetylation and transcriptional activation of p21, which promoted the differentiation of embryonic stem cells into endothelial cells^58^. In this study, we identified force-induced EZH2 reduction was critically implicated in the maintenance of super-enhancer stability. We found declined EZH2 expression was not associated with grossly reduced H3K27me3 occupancy but preferred loss surrounding strong H3K27ac peaks at the promoters of force-induced weakly changed genes and within SEs. This preference might reflect a selection pressure for the maintenance of active chromatin context at key regulatory elements including those within SEs and promoters of cell cycle and lineage specifying genes to avoid the otherwise abnormal de-repression at EZH2 tightly bound repressive regions. On the other hand, the ubiquitous boundary between SEs and H3K27me3 decorated domains supported a highly competitive antagonism between PRC2 and P300 to block the encroachment by the other one. Hence, EZH2 reduction is required to maintain the relative level of these two functionally opposing histone modifiers in mechanoresponse. Indeed, aberrant EZH2 compensation negatively affected H3K27ac levels and distribution within SEs which showed decreased H3K27ac deposition on average and loss of H3K27ac polarization at the constituent enhancers.

Decreased H3K27ac polarization and increased H3K27ac entropy of SEs observed in cells with the interference of force-induced EZH2 downregulation suggested a reduction of constituent enhancer specificity and a pertinent increase of degree of freedom for PRC2-associated and P300-associated substrate nucleosome competition, analogously reflecting an increased number of possible molecular configurations for PRC2 and/or P300 association with SEs in mechanoresponse. This explanation could link the increased H3K27ac entropy to augmented EZH2/P300 motions within SEs due to aberrant EZH2 compensation. However, it is technically difficult to directly measure EZH2 mobility under compressive force stress. Nevertheless, an alternative experiment examining EZH2 puncta formation and its co-localization with SE marker proteins provided some clues on this hypothesis. Indeed, in agreement with our proposition, increased spatial overlap between EZH2 and SE could be observed following mechanical stress exposure in PDLSCs with compensated EZH2 expression, suggesting an increased probability for EZH2 targeting onto SEs if the nuclear EZH2 concentration exceeded some threshold. Owing to the adjacency of SEs and H3K27me3 marked domains, we could envision that these spatial movements might promote random encounters between the two functionally opposing epigenetic domains.

Cytosolic EZH2 complex has been reported to interact with the VAV1 GEF, which facilitates the activation of Rho family GTPase Cdc42 and leads to actin polymerization and reorganization in cytosol^41,59^. In line with our observations that nuclear VAV pool exists in PDLSCs, VAV1 has been demonstrated to possess a nuclear localization sequence within its pleckstrin homology domain and show nuclear localization upon several extra-cellular or intra-cellular stimulations in immune cells^60,61^. The direct implications of all three VAV members in gene transcription regulation^61–63^ further highlighted a possible physical and functional interaction between nuclear VAV and epigenetic cofactors, such as EZH2. Importantly, although the precise molecular mechanisms of how mechanical stress is sensed and transduced to VAVs in PDLSCs are not clear, the critical involvement of GEF/RhoA pathway in mechanical response and cellular adaptation to force on integrins^64^, and the specific and key roles of VAV2 in mechanical stretch-induced RhoA activation in glomerular mesangial cells^65^, are consistent with the concerted actions of VAV-RhoA-cytoskeleton-EZH2 axis on epigenetic environment and chromatin structure in mechanotransduction in PDLSCs.

Given the crucial functions of super-enhancers in controlling the expression of cell identity genes^16,17^, it is reasonable to predict the deregulated differentiation potential of PDLSCs with preserved EZH2 expression after exposure to compressive stress owing to their SE destabilization. Indeed, we identified several critical lineage specifiers showing H3K27me3 signature and validated that PDLSCs with compensated EZH2 expression were deficient for lineage commitment into osteoblast, chondrocyte, and adipocyte. Actually, the multipotent PDLSCs could differentiate into a variety of lineages^66^ to replenish impaired cells during the healing of dental damages, and they have been demonstrated to be capable of generating a root/periodontal complex for the full support of normal tooth function in swine^67^. Therefore, on basis of the lamin/RB/E2F/EZH2 cascade mediated epigenetic remodeling of PDLSC, we could tailor the expressions or activities of lamin/RB/E2F/EZH2 axis by use of lamin and/or EZH2 pharmaceutical modulators^68,69^, in conjunction with carefully designed mechanical stimuli^70^ to optimize the conditions for periodontium regeneration and dental recovery as proposed in PDLSC based regenerative medicine^71,72^.

## Materials and methods

### Cell culture and treatment

The human samples were obtained at Peking University School and Hospital of Stomatology under approved guidelines set by Peking University Ethical Committee with informed donor consent. For mRNA expression analysis, we prepared PDLSC lines from eight donors^73,74^, and the RNA samples from one PDLSC line were subjected to RNA-seq. The PDLSCs used in this study were isolated and cultured according to previously reported protocols^73,74^. Briefly, the PDL tissues were separated from the mid-third of the root surface and then minced into small tissue cubes. Subsequently, the tissue cubes were digested with a solution of 3-mg/mL type I collagenase with 4 mg/mL dispase in α-minimum essential medium (α-MEM) for 15 min at 37 °C with vigorous shaking. The tissue explants were then plated into culture dishes containing α-MEM supplemented with 10% fetal bovine serum (FBS), 0.292 mg/mL glutamine, 100 units/mL penicillin streptomycin, and 100 μM ascorbic acid and incubated at 37 °C in a humidified atmosphere containing 5% CO_2_. The cells obtained were then subjected to flow cytometry analysis to examine the representative surface marks including CD45, CD34, CD29, CD90, CD73, and CD146, to validate the identity of isolated PDLSCs. Cells were tested negative for mycoplasma contamination and used at Passage 3–4 Static compressive force was applied to PDLSCs in vitro using the method previously described^26,75^. Briefly, a glass cylinder was placed over a confluent cell layer in the well of a 6-well plate. The compressive force was adjusted by adding lead granules to the cylinder. Cells were subjected to different continuous compressive forces ranging from 0 to 1.5 g/cm^2^ for 24 h or at 1.5 g/cm^2^ for different durations ranging from 0 to 24 h. In this study, we have used the glass cylinders which produce a compression of ~0.5 g/cm^2^ without lead granules in them. This compression strength makes the empty cylinder not suitable for work as negative control. As such, to control for the loading glass cylinder associated effects on medium contact and nutrient intake, we placed a light glass sheet with similar area but negligible weight on top of the control PDLSCs for comparisons with the compressive force stressed cells. Latrunculin B (Sigma) and CK666 (MedChemExpress) were used, respectively, to inhibit actin polymerization or activity of ARP2/3 complex.

### Quantitative reverse-transcription polymerase chain reaction (RT-qPCR)

Total cellular RNAs were extracted using Trizol reagent according to the manufacturer’s instruction. First strand cDNAs were synthesized with the Reverse Transcription System (Promega, A3500). Quantitation of all gene transcripts was done by quantitative PCR using Power SYBR Green PCR Master Mix and an ABI PRISM 7300 sequence detection system (Applied Biosystems, Foster City, CA) with the expression of GAPDH as the internal control. All the primers used for RT-qPCR are available upon request. To quantitatively test whether force could elicit a global amplification or suppression effect on gene expression, we took an external calibration strategy using the whole-cell spike-ins from Spodoptera frugiperda Sf9 cells, which was similarly described in a recent publication^76^. In this experiment, we performed RT-qPCR using a cell mixture with varying numbers of PDLSCs and fixed numbers of Sf9 cells. The expression levels of human genes of interest were then normalized to the levels of internal control genes from Sf9. This calibration method with an external reference point thus enables the estimate of absolute fold-change of human gene expression with respect to the number of cells in the sample.

### RNA-seq, ChIP-seq, and bioinformatics analysis

In-depth whole transcriptomic and ChIP sequencing were performed by the Beijing Genome Institute (BGI). The reads from RNA-seq were aligned to the human genome with TopHat^77^. We used Cufflinks^78^ to process the raw data and the gene expression level was represented by FPKM, which was normalized by the total number of transcripts per cell. Annotation for the differentially expressed gene sets were performed with Enrichr^29^. Gene Ontology analysis was performed by use of DAVID toolkits^79^. The reads from a ChIP-seq library were aligned to human genome with Bowtie^80^. Normalized ChIP-seq reads density were obtained through dividing reads coverage by the total number of mappable reads. We called super-enhancers by use of ROSE algorithm^17^ with default parameters for each H3K27ac ChIP-seq library. Epigenetic entropy of a SE was calculated as expected information content with respect to the probability mass function determined as normalized reads density along with each base within that SE. We used R (http://www.R-project.org) and ggplot2 packages (http://ggplot2.org) to produce heatmaps, and other statistical plots.

### Co-immunoprecipitation (Co-IP), western blotting analyses and indirect immunofluoresence (IF)

For Co-IP assay, cells were collected and lysed on ice with lysis buffer containing 0.5% NP40. The lysates were pre-cleared by incubation with protein A beads. The protein complex was then precipitated by a specific antibody together with protein A beads followed by extensive washing. The resulting materials were analyzed by western blotting as previously described^81^. For IF analysis, PDLSCs were fixed using 4% paraformaldehyde, permeabilized with 0.2% Triton X-100 and blocked with 4% BSA/PBS. Then the cells were treated with primary antibodies followed by incubation with FITC or TRITC–conjugated secondary antibodies. Coverslips were mounted in a glycerol/PBS solution containing DAPI. Confocal images were obtained using a Zeiss LSM confocal laser-scanning microscope. Antibodies against EZH2 (#5246), E2F1 (#3742), lamin A/C (#4777), H3K27me3 (#9733), H3K9me2 (#4685), H3K9me3 (#13969), RB and phospho-RB (S780, S795, and S807/811) (RB Antibody Sampler Kit #9969) were purchased from Cell Signaling Technology (CST). Antibodies against GAPDH (AC002) and α-Tubulin (AC007) were purchased from Abclonal. Antibody against CEMP-1 (ab134231) was from Abcam. Antibody against CAP (sc-53947) was from Santa Cruz. Antibodies used in IF were: EZH2 (612667, BD Pharmingen), H3K27ac (A7253, Abclonal), MED1 (A300-793A, Bethyl) and VAV2 (YT4864, Immunoway).

### Alizarin red staining, Alcian blue staining, and oil red O staining

PDLSCs were cultured in standard medium until they reached sub-confluence. The medium was then switched to osteogenic differentiation media (supplemented with 0.1 µM dexamethasone, 0.05 mM ascorbic acid 2-phosphate, and 10 mM glycerophosphate (Sigma-Aldrich)) or chondrogenic differentiation media (STEMPRO Chondrogenesis Differentiation Kit, Life Technologies), or adipogenic differentiation media (supplemented with 1 µM Dex, 0.25 mM isobutylmethylxanthine, 50 µM indomethacin, and 10 µg/ml insulin (Sigma-Aldrich)) for three weeks. The medium was changed every 3 days. Then cells were fixed and subjected to Alizarin Red staining, Alcian Blue staining, and Oil Red O staining, respectively, as described elsewhere^43,82^.

### Subnuclear fractionation

ERNFs and SNFs were separated as briefly described: cells were washed, scraped into TEN buffer (150 mM NaCl, 1 mM EDTA, and 40 mM Tris, pH7.4), collected by centrifugation, and resuspended in lysis buffer (10 mM Hepes, pH7.9, 10 mM KCl, 0.1 mM EDTA, 0.1 mM EGTA, 1 mM DTT, and 0.5 mM PMSF). After 15 min on ice, NP-40 was added to a final concentration of 0.5%. Lysates were centrifuged, and the nuclear pellet was collected, resuspended in 20 mM of ice-cold Hepes, pH7.9, 0.4 M NaCl, 1 mM EDTA, 1 mM EGTA, 1 mM DTT, and 1 mM PMSF, sonicated for 1 min, and vigorously vortexed at 4 °C for 15 min. The nuclear lysate was centrifuged at 15,000 × *g* for 45 min at 4 °C to obtain the supernatant (SNF) and the pellet (ERNF) that were resuspended in lysis buffer plus NP-40 (1% to a volume equal to that of the SNF). Total cell lysates run alongside SNFs and ERNFs were collected from parallel plates.

### siRNA transfection and viral infection

Chemically synthesized double-stranded siRNA against E2F1, EZH2, Lamin A, RB, p130, p107 were transfected alone or in combination into PDLSCs using lipofactamine RNAiMAX (Invitrogen). The siRNA sequences were as follows: siE2F1-1: GACGUGUCAGGACCUUCGU, siE2F1-2: CUGCAGAGCAGAUGGUUAU; siEZH2: AAGACTCTGAATGCAGTTGCT; siLamin A: GAAGGAGGAACTGGACTTCCA; siRB: GCGCUCUUGAGGUUGUAAU; sip130: tcagctgtagtcctcataa; sip107: CAAGCTAATAGTCACGTAT. The adenoviruses expressing ADV4-EZH2 and the control vector were produced by GenePharma (Shanghai, China).

### Statistical analysis

Statistical analysis was performed with R and Microsoft Excel. Data in Figs. 3 and 7 were presented as mean ± SD from 3 independent experiments and assessed by one-way analysis of variance (ANOVA).

## Supplementary information

Supplementary Methods

Supplementary Figure 1

Supplementary Figure 2

Supplementary Figure 3

Supplementary Figure 4

Supplementary Figure 5

## Data Availability

RNA-seq and ChIP-seq data were deposited at the Gene Expression Omnibus (GEO) database (http://www.ncbi.nlm.nih.gov/geo/) with accession number GSE109168.
