# Supplementary material for: EZH2 reduction is an essential mechanoresponse for the maintenance of super-enhancer polarization against compressive stress in human periodontal ligament stem cells
Source: Cell Death Dis. 2020 Sep 15;11(9):757. doi: 10.1038/s41419-020-02963-3 (PMC7493952; doi:10.1038/s41419-020-02963-3)
Supplement: Supplementary file 1 — Supplementary Methods [file 41419_2020_2963_MOESM1_ESM.docx]

**Supplementary methods**

Animals and Orthodontic Force Application

Three 7- to 8-week-old male C57BL/6 mice (Weitong Lihua Experimental Animal Center, Beijing, China) weighing 20 to 25g each were used in this study with no randomization or blinding. Experimental protocols were approved by the Animal Use and Care Committee of Peking University. Mice were anesthetized with pentobarbital sodium (100mg/kg, injected intraperitoneally) prior to surgery. Mechanical force was applied following a previously described method^1^. Briefly, a nickel-titanium coil spring (0.2 mm in wire size, 1 mm in diameter, and 1 mm in length; Smart Technology) was bonded between the maxillary right first molar and maxillary incisors by flowable restorative resin (3M ESPE). Approximately 30 g of force was applied^2^. The contralateral first molar, which was barely padded with resin, was used as the control. The mice were sacrificed by pentobarbital sodium overdose after seven days of force application. Immunohistochemical staining was performed with a two-step detection kit (Zhongshan GoldenBridge Biotechnology, Beijing, China) as previously described^3^.

1. Cao H, Kou X, Yang R, Liu D, Wang X, Song Y*, et al.* Force-induced Adrb2 in periodontal ligament cells promotes tooth movement. *J Dent Res* 2014, **93**(11)**:** 1163-1169.

2. Taddei SR, Moura AP, Andrade I, Jr., Garlet GP, Garlet TP, Teixeira MM*, et al.* Experimental model of tooth movement in mice: a standardized protocol for studying bone remodeling under compression and tensile strains. *J Biomech* 2012, **45**(16)**:** 2729-2735.

3. Kou XX, Li CS, He DQ, Wang XD, Hao T, Meng Z*, et al.* Estradiol promotes M1-like macrophage activation through cadherin-11 to aggravate temporomandibular joint inflammation in rats. *J Immunol* 2015, **194**(6)**:** 2810-2818.
