# Supplementary figures and images for: EZH2 reduction is an essential mechanoresponse for the maintenance of super-enhancer polarization against compressive stress in human periodontal ligament stem cells
Source: Cell Death Dis. 2020 Sep 15;11(9):757. doi: 10.1038/s41419-020-02963-3 (PMC7493952; doi:10.1038/s41419-020-02963-3)

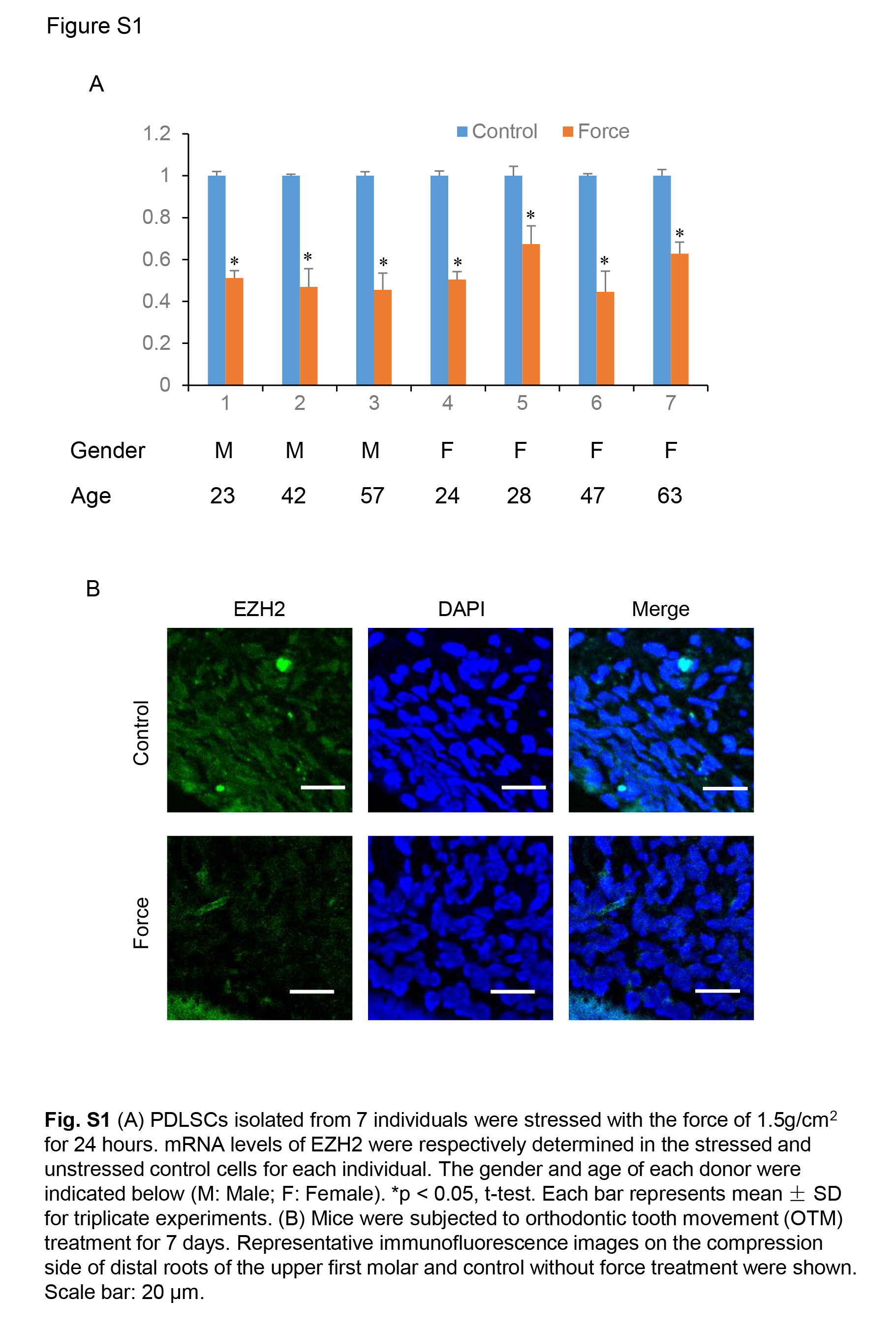

Supplement: Supplementary file 2 — Supplementary Figure 1 [file 41419_2020_2963_MOESM2_ESM.png]

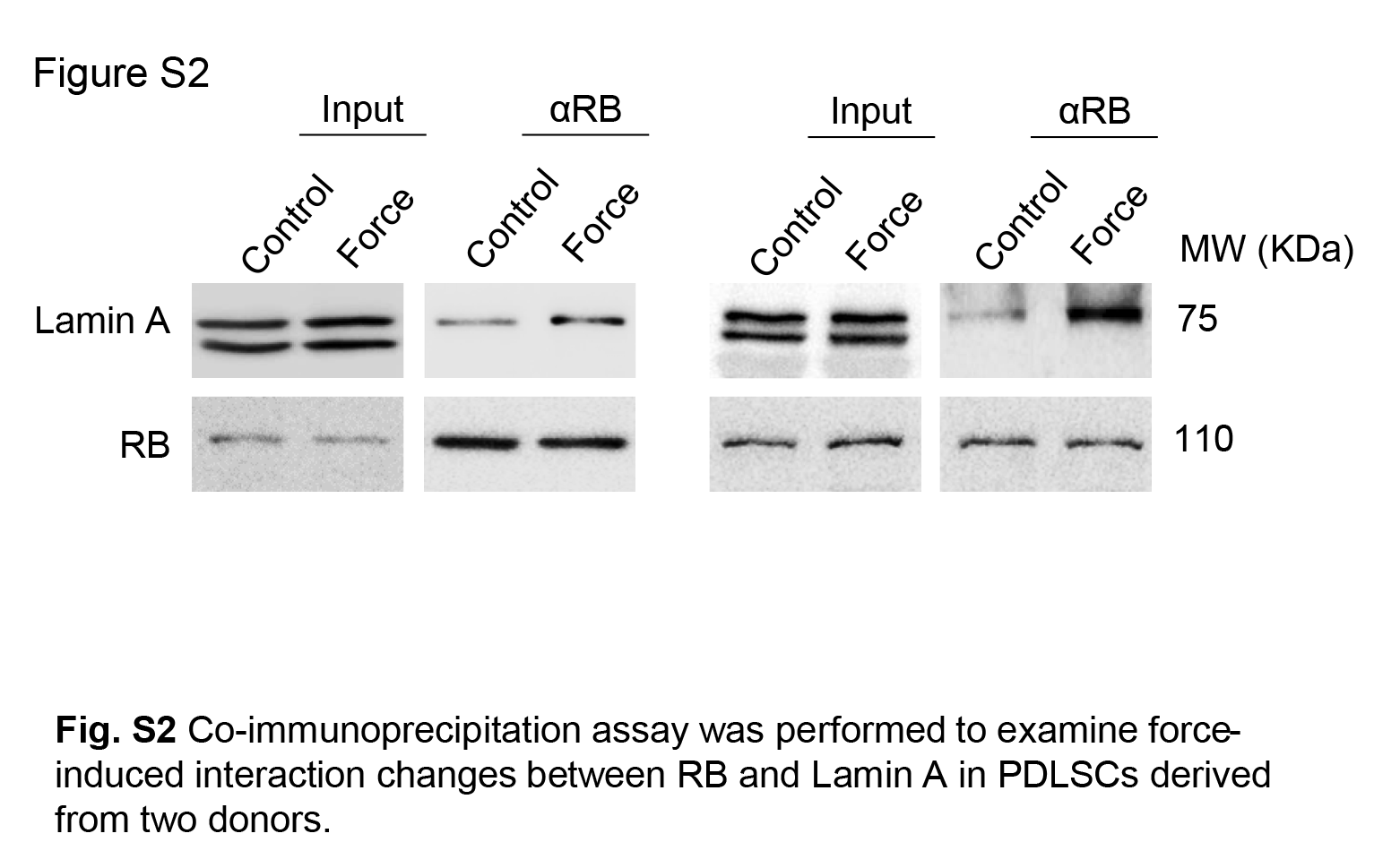

Supplement: Supplementary file 3 — Supplementary Figure 2 [file 41419_2020_2963_MOESM3_ESM.png]

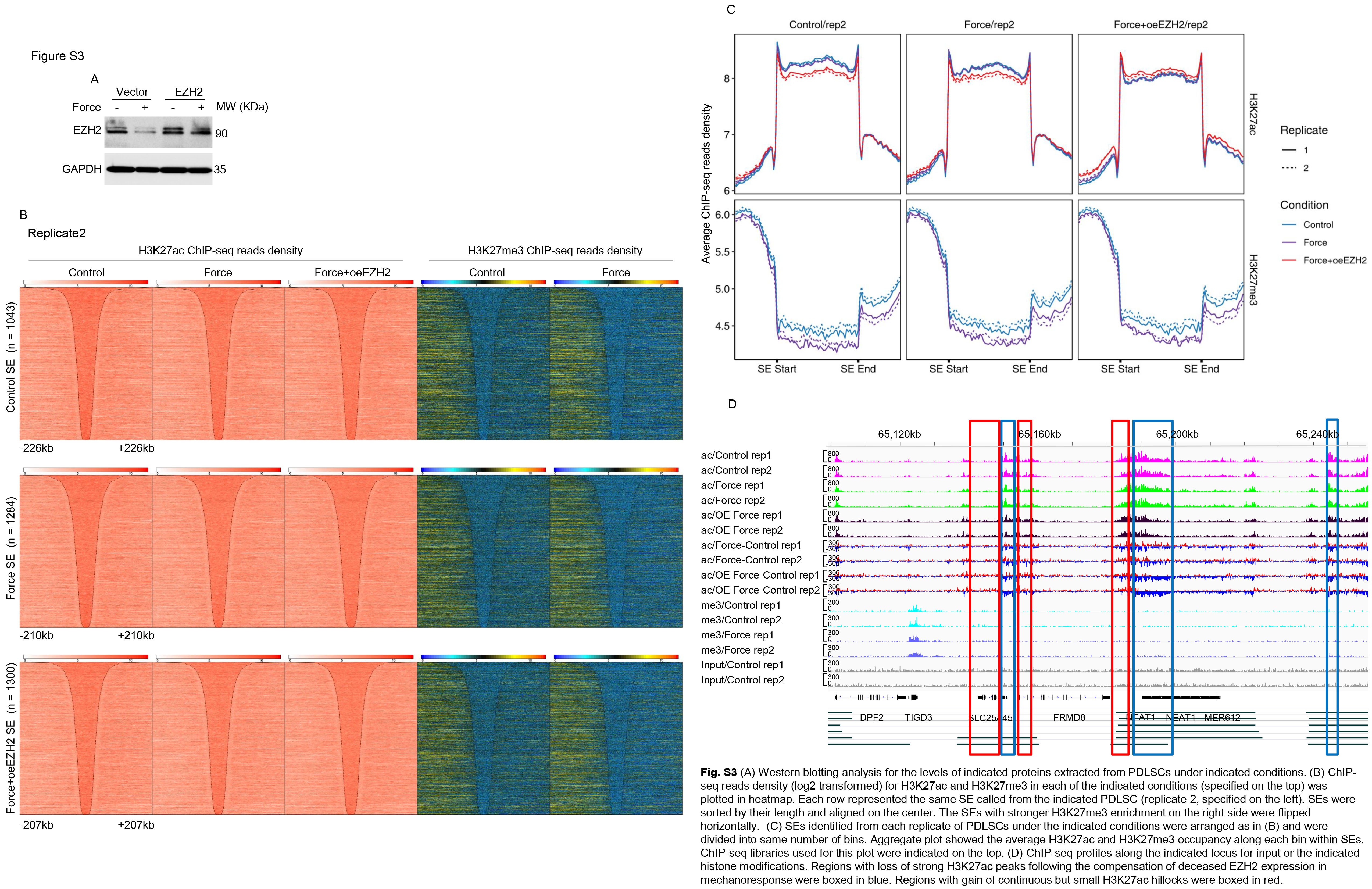

Supplement: Supplementary file 4 — Supplementary Figure 3 [file 41419_2020_2963_MOESM4_ESM.png]

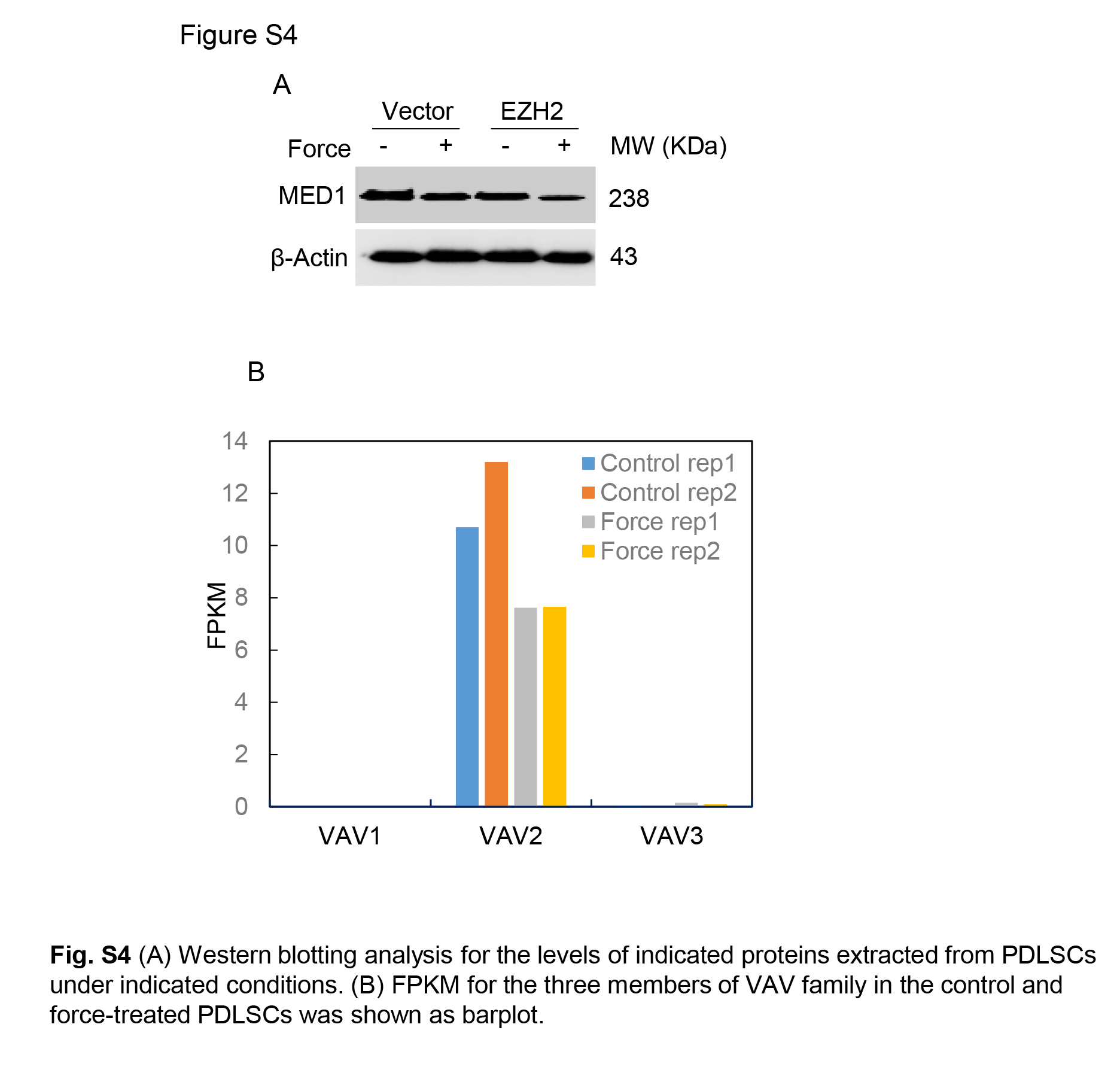

Supplement: Supplementary file 5 — Supplementary Figure 4 [file 41419_2020_2963_MOESM5_ESM.png]

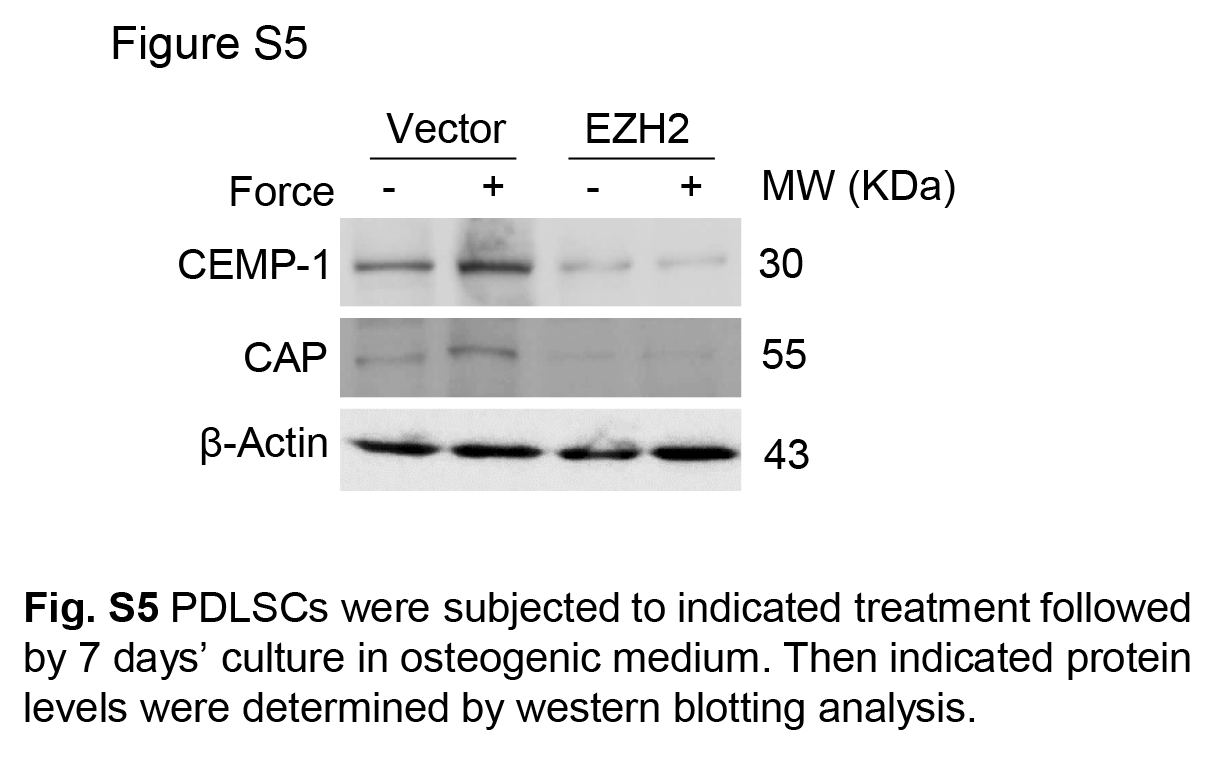

Supplement: Supplementary file 6 — Supplementary Figure 5 [file 41419_2020_2963_MOESM6_ESM.png]
